# Paediatric Partial-Thickness Burn Therapy: A Meta-Analysis and Systematic Review of Randomised Controlled Trials

**DOI:** 10.3390/life12050619

**Published:** 2022-04-21

**Authors:** Aba Lőrincz, Alex Váradi, Péter Hegyi, Zoltán Rumbus, Máté Tuba, Anna Gabriella Lamberti, Margit Varjú-Solymár, Andrea Párniczky, Bálint Erőss, András Garami, Gergő Józsa

**Affiliations:** 1 Department of Thermophysiology, Institute for Translational Medicine, Medical School, University of Pécs, 12 Szigeti Street, H7624 Pécs, Hungary; aba.lorincz@gmail.com (A.L.); zoltan.rumbus@aok.pte.hu (Z.R.); mate.tuba@gmail.com (M.T.); lambi.anna.gabi@gmail.com (A.G.L.); andras.garami@aok.pte.hu (A.G.); 2 Department of Biostatistics, Institute for Translational Medicine, Medical School, University of Pécs, 12 Szigeti Street, H7624 Pécs, Hungary; varadi.alex@pte.hu; 3 Institute for Translational Medicine, Szentágothai Research Centre, Medical School, University of Pécs, 20 Ifjúság Street, H7624 Pécs, Hungary; hegyi.peter@pte.hu (P.H.); margit.solymar@aok.pte.hu (M.V.-S.); andrea.parniczky@gmail.com (A.P.); eross.balint@pte.hu (B.E.); 4 Centre for Translational Medicine, Semmelweis University, 26 Üllői Street, H1085 Budapest, Hungary; 5 Division of Pancreatic Diseases, Heart and Vascular Center, Semmelweis University, 68 Városmajor Street, H1124 Budapest, Hungary; 6 Division of Surgery, Traumatology and Otorhinolaryngology, Department of Paediatrics, Clinical Complex, University of Pécs, 7 József Attila Street, H7623 Pécs, Hungary

**Keywords:** paediatric second-degree burn, silver sulphadiazine, silver foam, biosynthetic dressing, skin substitutes

## Abstract

Background: Paediatric second-degree burn injuries are a significant source of medical challenges to the population that may cause severe, lifelong complications. Currently, there are dozens of therapeutic modalities and we aimed to summarise their reported outcomes and determine their effectiveness, compared to the widely used silver sulphadiazine (SSD). Methods: We conducted the meta-analysis and systematic review of randomised controlled trials (RCTs), which investigated the performance of dressings in acute paediatric partial-thickness burns. The evaluated endpoints were time until wound closure, grafting and infection rate, number of dressing changes and length of hospitalisation. Results: Twenty-nine RCTs were included in the qualitative and 25 in the quantitative synthesis, but only three trials compared SSD directly to the same intervention (Biobrane). Data analysis showed a tendency for faster healing times and a reduced complication rate linked to biosynthetic, silver foam and amnion membrane dressings. A substantial difference was found between the number of dressing changes associated with less pain, narcosis and treatment duration. Conclusions: Considerable between-study heterogeneity was caused by the unequal depth subcategory ratio and surface area of the injuries; therefore, no significant difference was found in the main outcomes. Further research is necessary to establish the most effective treatment for these burns.

## 1. Introduction

Nearly one hundred thousand (viz., ~96,000) children suffer a fatal injury from preventable, mostly flame-related (~93%) burn traumas, each year, that is 263 cases per day, according to the WHO’s latest global report. The likelihood of a non-fatal injury is assumed to be at least ten times higher (i.e., 1–7,000,000/year), and due to the absence of a successfully coordinated prevention, treatment or rehabilitation strategy, almost half of them (49%) suffer from some form of irreversible disability after the burn [1,2]. Complications such as extensive contractures and amputations constitute physical impairments, but even a relatively minor scar or the memory of the trauma can provoke lifelong psychological disorders [3,4,5]. Compared to adults, children, especially infants, have thinner skin and immature defensive reflexes, with limited environmental experience in addition to their natural curiosity towards their surroundings [6,7,8]. Therefore, by touching hot surfaces and pulling hot liquids onto themselves, children can severely injure often multiple and critical parts of their body: upper limbs, head and neck, and legs in 51, 39 and 26% of all cases, respectively [9].

The severity and prognosis of a burn are determined by the depth, area and location of the injury, along with the patient’s general health and age. Burns are often mixed depth, in a map-like pattern; thus, evaluating their exact severity still constitutes a challenge [10]. In second-degree or partial-thickness burns (PT or II), the skin’s dermis layer is affected, and it can be further classified into two subcategories. Superficial partial-thickness thermal injuries (II/A) involve the papillary layer of the dermis. In II/A burns, spontaneous healing takes on average 7 to 10 days and long-term pigmentation changes may occur. Straw-yellow bullae and—after their removal—painful, moist, bright pink wound beds with intact epidermal appendages characterise this condition.

In comparison, deep PT burns (II/B) damage the stratum reticulare as well, and the wound bed turns numb and dry with a blotched pale, white or purple colour and the loss of all epidermal appendages. Spontaneous recovery often results in extensive hypertrophic scar development and contractures. In full-thickness (third-degree or III) thermal injuries, the entire skin is necrotised and becomes leathery dry, painless, as well as pale, and pearly [11]. Complete regeneration does not occur by primary intention, and an operative approach is necessary to help these patients [12].

While advances in medicine have led to the introduction of an abundant number of therapeutic options to treat children with PT burns, many questions remained unanswered regarding their optimal use and effectiveness. The different interventions were primarily developed for the chronic wounds of adult patients, while paediatric burn injuries possess different healing potential qualities, inflammatory status and exudation [10]. Some dressing materials may be better suited for treating burns in younger patients because of their different burn aetiology, physiology and still evolving nature [13]. In the case of delayed or inadequate medical interventions, the frequency, severity and duration of the complications are increased, resulting in extended hospital stays and the higher use of anaesthetic and analgesic drugs, as well as the total cost of care. Therefore, a rapid and effective therapeutic response is critical in these severe forms of burns (i.e., II/B, III) [12]. At the same time, the lack of current evidence-based treatment guidelines makes it hard to determine which materials should be preferred for a specific type of injury.

The management of paediatric PT burns consists of primary care (e.g., cooling, painkillers, fluid resuscitation and transportation), cleaning and disinfecting the wound, then removing the necrotic tissue. After that, the surgeon must restore the damaged skin barrier to protect the patient from fluid loss and infections. The burn wound is either covered in a conservative approach with dressings and topical ointments or surgery is performed by sewing a skin graft onto the injury site followed by the application of a conservative dressing [12,13,14]. Recent studies confirmed that a moist environment is beneficial for burned tissue regeneration [15]. The ideal temporary skin replacement possesses absorbent and antimicrobial qualities, can be quickly and painlessly changed—so it must not stick to the wound bed—, while it also stays in place during the healing of the wound. It should be transparent as well—to be able to monitor the injury—, and affordable, without causing any irritation or toxicity. Such an ideal dressing, which fulfils all these criteria, unfortunately, does not yet exist, but certain interventions’ attributes are closer to the idyllic model than others.

In the past, the gold standard for the topical treatment of paediatric PT burns was the soft, white and water-soluble silver sulphadiazine (SSD) 1% cream, under many product names such as Dermazin^®^, Flamazine^®^, Silvadene^®^ or Silvazin^®^ [16,17,18,19,20,21,22,23,24,25,26,27]. It is still the most commonly administered treatment in many countries as it allows wounds to heal without the need for surgical intervention. Thus, we chose this therapy as a comparator because most of the articles reported their findings correlated to SSD due to its historical relevance. However, numerous studies revealed several disadvantages to the use of SSD, which led to the development of a wide range of alternative topical treatments. Nevertheless, their efficacy in the management of paediatric PT burns remains largely unclarified. A summary of each intervention that was analysed and compared to SSD in the present study can be found in Text S1.

We performed a literature search to systematically review the available treatment options for paediatric PT burns, then conducted a meta-analysis to obtain insights into the dressings’ healing potential and complication rate. We aimed at collecting randomised controlled trials (RCTs) about PT burn treatments that reported the time to reepithelialisation (TTRE), grafting and infection rate, number of dressing changes and length of hospital stay (LOS) in patients younger than 18 years old at the time of the injury.

## 2. Materials and Methods

### 2.1. Search Methods for Identification of Records

**Figure 1:**
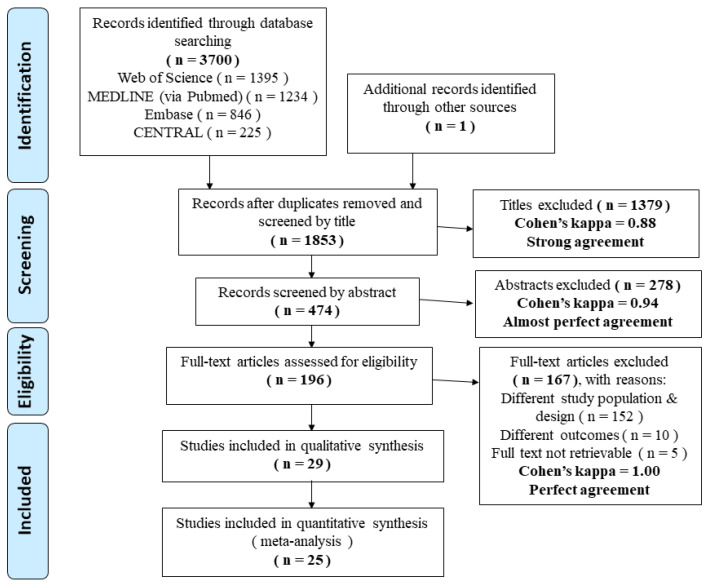
PRISMA flow chart. It represents the process of the study search and selection.

On 29 October 2020, a systematic search was conducted in accordance with the Preferred Reporting Items for Systematic Reviews and Meta-Analysis Protocols (PRISMA) (Table S1) [28]. We searched for RCTs that compared at least two different interventions in patients under the age of eighteen with PT burns in the MEDLINE (via PubMed), Embase, Web of Science and CENTRAL databases (Figure 1).

Our search keys can be found in Text S2, and the records identified by them were exported without the use of filters.

### 2.2. Study Selection, Data Extraction and Management

The studies identified by the search were screened by two independent review authors (AL and MT) to assess their eligibility. The eligible articles were collected in EndNote X9 (Clarivate Analytics, Philadelphia, PA, USA), then the outcomes recorded in Microsoft Excel (Microsoft Corporation, Albuquerque, NM, USA) by two authors independently (AL and MT). Discrepancies were resolved by consensus after re-checking the original article.

The extracted data consisted of the children’s characteristics, including the number of participants, age, depth of injury, percentage of burned area compared to total body surface area (TBSA%), and the type of interventions, as well as the reported outcomes, such as TTRE, grafting rate, infection rate, number of dressing changes and LOS. Further parameters, such as treatment cost, pain and scarring could not be analysed among the outcomes because of data ineligibility.

### 2.3. Assessment of Methodological Quality of Included Records

The risk of bias of the individual RCTs was assessed as “low”, “some concerns” or “high”, independently by the two investigators (AL and MT) with the use of the Cochrane Collaboration’s RoB2.v7 tool. Discrepancies were resolved by consensus. Randomisation process, deviations from the intended interventions, missing outcome data, measurement of the outcome and the selection of the reported results were evaluated to conclude the overall bias of each article.

Additionally, the evaluation of funding sources, conflict of interest statements and adherence to the Consolidated Standards Of Reporting Trials (CONSORT) statement were also conducted, using which, criteria were developed in order to ascertain the standardization and reproducibility of the RCTs [29].

### 2.4. Data Synthesis

Statistical analysis was performed by an expert biostatistician (AV) using the methods recommended by the working group of the Cochrane Collaboration [30]. In the meta-analysis, the calculated effect sizes were visualised in forest plots using Comprehensive Meta Analysis (Version 3) statistical software (Biostat Inc., Englewood, NJ, USA). Heterogeneity was tested with Cochrane’s Q (χ^2^) test and the I^2^ statistic. Q test was considered significant when *p*-values were less than 0.1. Based on the suggestion of the Cochrane Handbook, I^2^ values from 30% to 60% represent moderate and between 50% and 90% substantial heterogeneity. Due to the groups’ generally high heterogeneity, DerSimonian and Laird random-effects models were used in all analyses [31].

For continuous outcomes: means, and for dichotomous outcomes: event rates with 95% confidence intervals, were pooled in each subgroup to compare the differences between the intervention groups. In the case of some subgroups, there were studies with more than one intervention group; therefore, we combined these groups based on the suggestion of the 6.5.2.10 section of the Cochrane Handbook [30]. When the means and standard deviations (SD) of the effects were not reported, we derived these data from the graphical representation of the outcomes or by estimation based on the work of Wan et al. 2014 with the use of medians, minimum, maximum or quartiles [32]. In three trials, indicators of SD were not reported [21,22,23], thus, we obtained them from a previous meta-analysis [33], which included the required data.

## 3. Results

### 3.1. Search Results

The search identified 1853 potentially relevant records after duplicate removal that were screened by title (Figure 1). After exclusion, 474 abstracts were assessed. The full texts of a total of 196 articles were retrieved; then, 152 trials were excluded because they had an unmatching or unknown study population or design. Ten studies did not contain the specified outcomes and five full texts could not be obtained; thus, these were also excluded from the analysis. Finally, 24 RCTs containing 21 full-text articles and 3 conference abstracts [27,34,35] were included in this meta-analysis. Another full-text article [26] was identified when the reference lists of the eligible papers were checked; it was included in the qualitative synthesis. In the systematic review, an additional three full-text RCTs [36,37,38] and one conference abstract [39] were included.

### 3.2. Description of Included Studies

SSD [16,17,18,19,20,21,22,23,24,25,26,27] treatment was reported in comparison with amnion membrane (AM) [20,40,41], biosynthetic dressings (Biobrane, EzDerm, Transcyte) [18,21,22,40,41,42,43,44], Biobrane only [18,21,22,42,43], negative pressure wound therapy (NPWT) [27,35,45], silver foam dressings (Acticoat, Aquacel Ag, Mepilex Ag) [43,44,45,46,47,48,49,50,51] and Acticoat only [46,47,49], which can be seen in the following figures. The attributes of each aforementioned intervention, as well as autografts [41,52], Silvasorb [25], Tilapia [16], additional treatments (viz., collagenase [19], vitamin E + C + Zinc [53], wIRA [36], heparin [38], rhGM-CSF [34], bFGF [37] and rhEGF [39]) and combination therapies (Acticoat + Mepitel [45,46,50,51], NPWT + Acticoat + Mepitel [45], Biobrane + Acticoat [26,49], Biobrane + Recell [43]) are summarised in the following tables and Tables S2–S6. Only one multi-centre study [17] was identified. Six studies reported outcomes from II/A [16,20,22,35,36,39], five from II/B [20,27,34,39,41], three from II/A and MD [17,25,42] and one from MD injuries only [46], while in the remaining studies, the exact depth of the injury was not specified in children with PT burns [18,19,21,23,24,26,37.40,43–45,47,48,53]. The trial and patient characteristics of the 29 RCTs analysed in this study are summarised in Table 1.

The mean age of the patients was 4.3 years. Of 756 patients, 14.3% were younger than one year, 78.6% were below the age of five and 21.4% were older than five years. The majority of the patients were boys: 655 out of 1089 children (59.1%). The 832 patients had an average of 7.5 TBSA%, which was distributed among the children as follows: 23.2% under 5 TBSA%, 46% between 5–10 TBSA% and 30.8% above 10 TBSA%. It is important to highlight that six articles did not report TBSA% [17,25,34,35,36,39]. Moreover, two studies reported median TBSA% without appropriate indicators of SD, thus, proper conversion from median to mean was not possible. Five trials included exclusively scalds [22,23,35,43,48]. In the remaining studies, the aetiological distribution of 628 patients’ burns were 65.5% scalds, 18.7% flame, 15.4% contact and 0.5% electrical injuries. In most articles, the TTRE, grafting and infection rates, the number of dressing changes and LOS were assessed as outcome parameters. The TTRE was not discussed in only three trials [19,41,47], while the other parameters were reported in various fashions.

### 3.3. Methodological Quality of the Included Studies

A summary of the risk of bias assessment is shown in Figure S1. Generally, the risk of bias was considered high, and the articles often lacked essential information. Randomisation protocols were generally not discussed, but studies reported the use of lottery [16,21], tables of random numbers combined with lottery [20] and a randomization schedule [34]. Computer-generated individual unit block randomization [19] and randomization tables [27,35,47,49]—including one that was further stratified by age and area [17]—along with a statistician generated age-stratified permutated block method [45] were also used. One article divided the treatment groups by even and odd admission days [37], and one study contained seven patients with resident preference-based randomisation in addition to a computer-generated randomisation table [22].

Only seven articles mentioned allocation concealment with opaque, sealed envelopes [43,47], sealed envelopes [44], externally created coded envelopes [43,48], burn area stratified sealed envelopes [26], computer-generated results [47], REDCap concealment [45] or by not making them available to the caregivers [19]. Most of the studies could not be blinded due to the interventions’ distinctive qualities, but there was one patient-blinded [16], three assessor-blinded [45,46,47] and five double-blind studies [26,36,39,41,53]. Selective reporting was challenging to estimate because only six articles referred to their original trial protocol [19,26,37,43,46,48].

While evaluating the funding sources, we found that eight articles received either financial or material donations from the manufacturer [17,20,21,25,45,46,53] and two were supported by solely independent grants [37,47], although most of these researchers stated that they had no conflict of interest, with two exceptions. One of the funders supervised the design of the study, the data analyses and the development of the manuscript [17], and another intervention was developed by the first author, who is also the director of the company that sells it [43].

Overall, two studies reported using the CONSORT criteria while conducting the research [45,46], which may be the reason behind the missing data, such as randomisation or concealment protocols.

### 3.4. Effects of Interventions

#### 3.4.1. Time to Reepithelialisation (TTRE)

**Figure 2:**
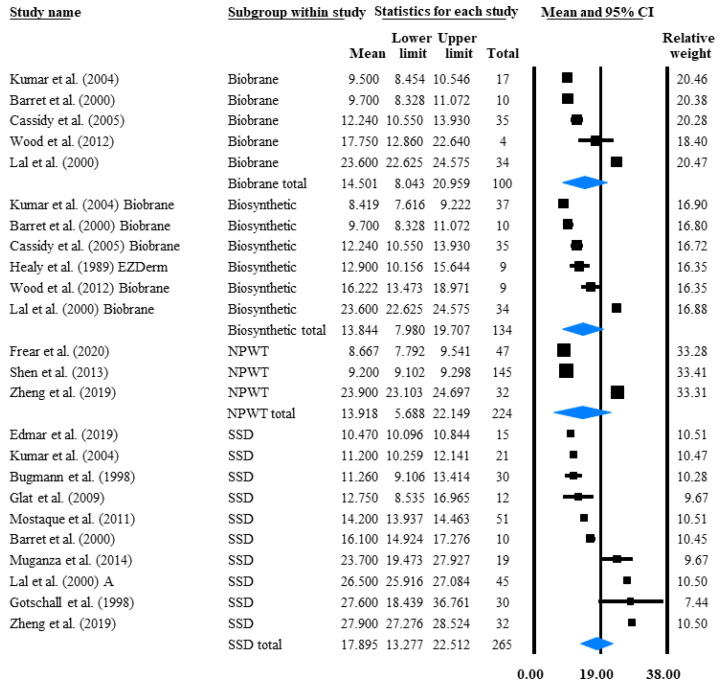
Average wound closure time. This forest plot of studies pools mean TTRE intervals in days, lasting from the time of the paediatric PT burn injury until wound closure. Black squares indicate the TTRE in each study. The size of the black squares represents the individual study weight, and the horizontal lines show their corresponding 95% confidence intervals (CIs). A blue diamond indicates the overall effect, and its outer edges characterise the Cis [16,18,20,21,22,23,24,25,26,27,35,42,43,44,45].

Our primary outcome to determine the interventions’ effectiveness was the mean TTRE or complete wound closure time. A total of 623 participants (ranging from 4 to 145 in the different studies with an average of 30) from 17 trials were included in this meta-analysis. Interventions with similar characteristics were pooled together to rank this outcome because direct comparisons were only published for SSD and Biobrane in a sufficient quantity. In total, 265 children received SSD with a mean TTRE of 17.89 days, which was the slowest among the analysed interventions, although the difference was not statistically significant (*p* = 0.70). Lower TTRE was seen in 224 children treated with NPWT (13.92 days) and in 134 patients receiving biosynthetic dressing (13.84 days), out of which 100 children were treated with Biobrane only (14.5 days) (Figure 2).

Further analysis was conducted to find out the reason behind the groups’ considerable heterogeneity (which was indicated by a high I^2^ of 75.35–99.85). Not surprisingly, when mean TTRE was stratified by depth, a significant difference (*p* = 0.0004) was found between II/B (20.53 days), II/A (13.77 days) and combined PT (12.43 days) burns (Figure S2). This difference also clearly indicates that PT burn subcategories should be analysed separately, even though most of the articles [18,19,21,23,24,26,37,40,43,44,45,47,48,53] and a previous review [33] pooled them together. Due to the low number of eligible studies in each subgroup, we were not able to conduct a meta-analysis on the individual intervention’s TTRE stratified by the depth of the burn. Nevertheless, we pooled and ranked the treatment options according to their depth; in II (Table 2), II/A (Table S2), II/A + MD (Table S3) and II/B (Table S4) PT burns.

We classified the TTRE by the affected surface area as well (<5 TBSA%: 13.16; 5–10 TBSA%: 16.07; 10–25 TBSA%: 16.20 days) (Figure S3). However, as a result of uneven depth subcategories, no significant difference was found (*p* = 0.77).

Another strong correlation between the burn area and TTRE was observed when we developed a novel ratio of TBSA% to TTRE (T%/T), which indicates what percentage of the TBSA regenerates each day and can also be used to standardise the burn sizes (Table 2 and Tables S2, S4 and S5). A critical limitation of using T%/T is that in smaller (under 5 TBSA%) burns, the ratio will be low, even though reepithelialisation was rapid. The reason behind this, in our hypothesis, is that there seems to be a minimum physiological time for wound regeneration, which is unrelated to the burn size and takes approximately 5–7 days. In studies that did not report TBSA% [17,25,34,35,36,39] or TTRE [19,41,47], the T%/T ratio could not be calculated (Table S3). Since TTRE alone seems insufficient to determine the additional interventions’ effectiveness on wound closure (e.g., vitamins or heparin), we also calculated these therapies’ TTRE reduction percentage (TTRE red%). The additional interventions were compared to their control treatment, where they received a placebo (or nothing) instead, on top of the traditional treatments (Table 2, Tables S2 and S4).

For SSD only, TTRE was also compared in subgroups divided by depth (SSD II: 17.11; SSD II/A: 17.05 days; *p* = 0.99) and by area (<10 TBSA%: 17.03; 10–25 TBSA%: 20.59 days) (Figures S4 and S5), but the differences were not significant between the subgroups (*p* = 0.59). This may indicate that the burns categorised as PT were mostly II/A injuries. Some articles reported the fraction of wound closure on the tenth day (day 10 RE%), which is summarised in Table S5.

Only three trials [18,21,22] reported the TTRE of the same comparator (SSD: 17.94 days) and intervention (Biobrane: 14.27 days), which were analysed separately. Despite the lack of a significant difference between the two treatments (*p* = 0.61), every article reported improved results with Biobrane compared to SSD (Figure S6).

#### 3.4.2. Grafting Rate and Non-Grafted Rate

**Figure 3:**
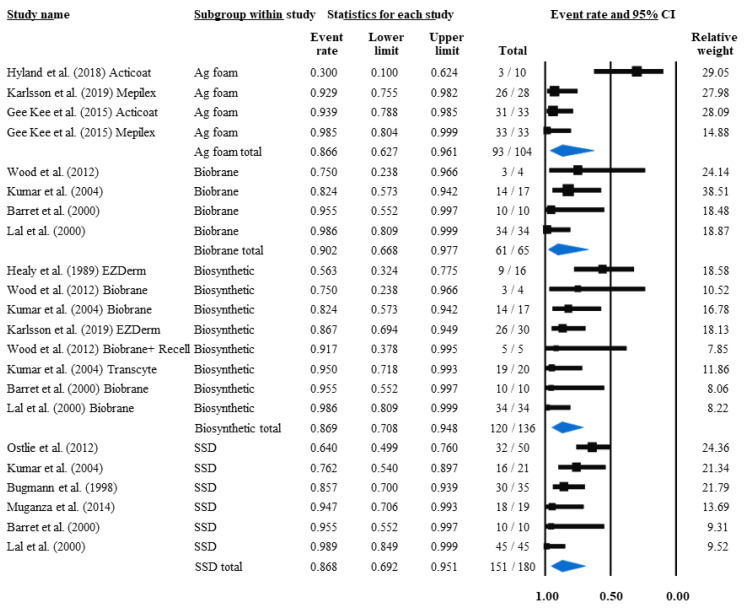
Mean non-grafted population ratios. They should be interpreted as grafting rates of paediatric PT burns when subtracted from 1 (100%). Black squares indicate the TTRE in each study. The size of the black squares represents the individual study weight, and the horizontal lines show their corresponding 95% confidence intervals (CIs). A blue diamond indicates the overall effect, and its outer edges characterise the CIs [18,19,21,22,24,26,43,44,46,48,49].

If conservative treatment is unable to heal the injury, a permanent skin transplantation is needed to facilitate wound closure. First, the interventions’ mean percentages of how many conservatively treated patients required grafting related to the whole study population were calculated. The ratio of grafted patients was 19.3% in SSD-, 20.5% in biosynthetic-, and 18.9% in silver foam-treated patients (*p* = 0.99) (Figure S7). Because every treatment without any grafted patient (zero outcomes) had to be excluded from the previous meta-analysis, here we used the reverse approach; that is, the comparison of the percentage of the non-grafted patients among the treatments (Figure 3).

With this method, we found that by subtracting the non-grafted population percentages from 100%, the grafting rate was 13.2%, 13.4%, 13.1% and 9.8% in patients treated with SSD, silver foam, biosynthetic and Biobrane, respectively. These results indicate that among Biobrane-treated children, grafting was required 25.8% less often compared to SSD; however, the difference between the treatments did not reach the level of significance (*p* = 0.98).

Similarly to TTRE, the grafting rate for SSD (7.6%) and Biobrane (6.6%) was analysed separately in the three articles that compared both of them (Figure S8) [18,21,22], which showed a 13.2% reduction in grafting need (*p* = 0.92) in Biobrane-treated children compared to SSD. The specific intervention analysis revealed therapeutic options that may result in reduced grafting rates, which were Transcyte (5%), Mepilex Ag (3.3%), NPWT + Acticoat + Mepitel (2.1%), and Biobrane + Recell (0%), but due to the scarcity of data, the statistical analysis to detect a significant difference could not be performed (Table 3).

#### 3.4.3. Dressing Changes

There was not enough data to conduct a meta-analysis of the required dressing changes between the two interventions. Nevertheless, the mean frequency of dressing reapplications showed a great variance among interventions, and they positively correlated with pain and discomfort levels. Furthermore, dressing changes were proportional to the rate of anaesthesia induction as well as to the time required for the healthcare professionals and the operating theatre for the administration of the treatments. SSD seemed to be the least efficient option with an extremely high 65.5 mean dressing changes if the wounds were treated openly and 9.6 dressing changes with closed wound treatment. Interventions with three or fewer dressing reapplications were Acticoat + Mepitel and Tilapia (number of changes 3.0 for both), Acticoat (2.7), NPWT + Acticoat + Mepitel (2.4), Transcyte (1.5), AM alone (1.3) or with nystatin and polymyxin B (PMB) (0.5), and Aquacel Ag (1.0) (Table 4).

#### 3.4.4. Infection Rate and Non-Infected Rate

**Figure 4:**
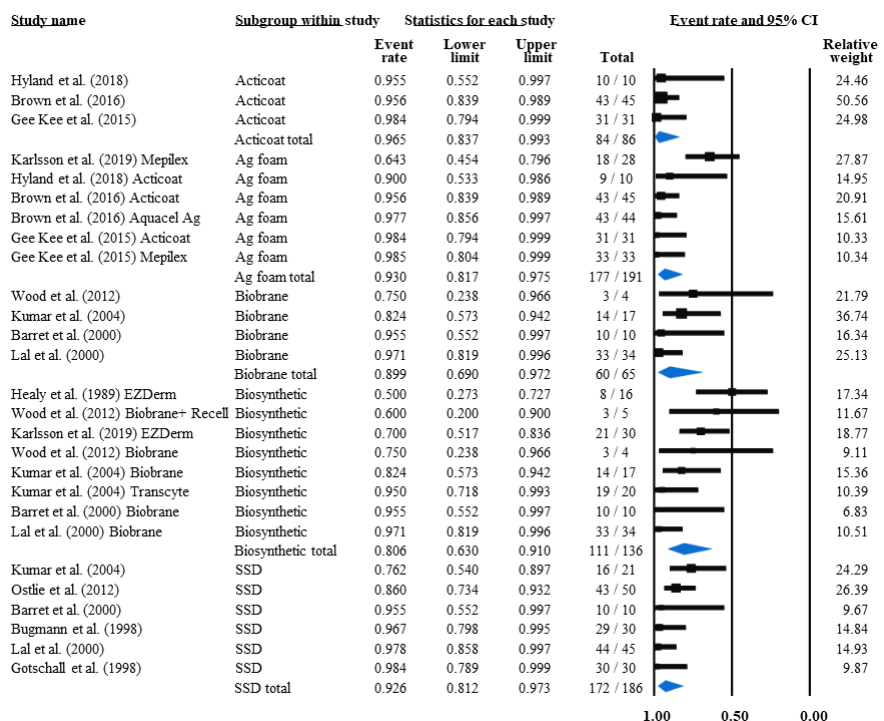
The average non-infected population rates. Five paediatric PT intervention groups’ antimicrobial effectiveness was compared. The results should be interpreted as infection rates when subtracted from 1 (100%). Black squares indicate the TTRE in each study. The size of the black squares represents the individual study weight, and the horizontal lines show their corresponding 95% confidence intervals (CIs). A blue diamond indicates the overall effect, and its outer edges characterise the CIs [18,19,21,22,23,24,43,44,46,47,48,49].

The patients’ percentage that showed signs of infection during their treatment was calculated similarly to grafting needs. The infection rates in the cases of different interventions were as follows: biosynthetic dressings: 21.8%; silver foam dressings: 12.4%; Biobrane: 11.7%; SSD: 9.2% (*p* = 0.65) (Figure S9). By subtracting the non-infected children’s percentages from the whole population, the calculation revealed slightly different results. In this case, biosynthetic dressings still had the highest microbial contamination rate of 19.4%, among which the rate for Biobrane was 11.7%. SSD showed an even lower rate of 7.4% infections, while the percentage of infected patients was 7.0% in silver foam dressings and 3.5% in Acticoat treatment groups (*p* = 0.24) (Figure 4).

Individual intervention effect analysis indicated potential alternatives with more advantageous effects on infection rates, such as Aquacel Ag foam (2.4%) and PMB combination therapies such as collagenase or AM with Nystatin (2.0% and 1.9%, respectively) or Acticoat with Mepitel (0%) (Table 5).

Additionally, the infected population rate in the case of SSD (13.9%) was similar to the rate of Biobrane (9.9%), without a significant difference (*p* = 0.91) between the treatments (Figure S10).

#### 3.4.5. Length of Stay (LOS)

**Figure 5:**
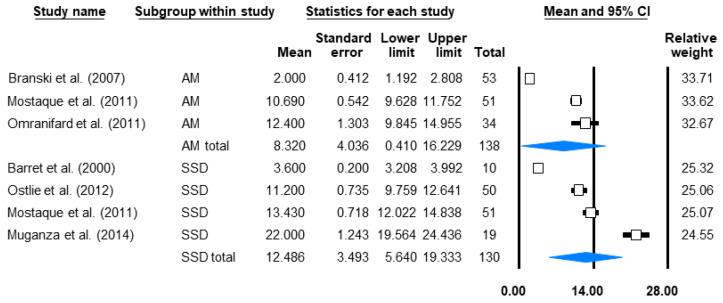
Average length of stay. The days spent inside the hospital by children treated with SSD or AM for PT burns. Black squares indicate the TTRE in each study. The size of the black squares represents the individual study weight, and the horizontal lines show their corresponding 95% confidence intervals (CIs). A blue diamond indicates the overall effect, and its outer edges characterise the CIs [18,19,20,26,40,41].

The length of hospital stay—the time spent inside the hospital from admission until discharge—is associated with the total cost of care, and it enormously impacts the children’s discomfort levels. Sufficient data for a meta-analysis was only available for SSD- and AM-based treatments, for which treatments the mean LOS were 12.5 and 8.3 days, respectively (Figure 5).

While this indicates a 33.6% shorter LOS in the case of AM-based compared to SSD-based treatments, the difference was non-significant (*p* = 0.43). The analysis of specific interventions showed that without antibiotic coverage, LOS is similar in the case of amniotic membrane and SSD treatments (11.37 vs. 13.77 days), while the addition of nystatin and PMB can reduce LOS to 2 days (Table S6). It is important to note that in the cases of several treatments, such as EZDerm (LOS: 3.4 days), Mepilex (LOS: 3.1 days), Biobrane (LOS: 2.4 days) and AM + Nystatin + PMB (LOS: 2 days), the children could be discharged even before the complete reepithelialisation of their injuries, whereas patients treated with SSD and collagenase stayed in the hospital for the entire duration of dressing changes.

## 4. Discussion

Even though SSD is widely used as a treatment for burns, our study concluded that it has some disadvantages that can outweigh its beneficial effects, which are mainly its applicability, low cost and notable antibacterial efficacy (i.e., an infection rate of 9.22%) [16,17,18,19,20,21,22,23,24,25,26,27]. However, SSD was associated with slow wound closure (TTRE II/A: 11.0 days; II/B: 25.7 days; II: 18.3 days and 0.39 T%/T) and prolonged hospital stay (LOS II: 13.8 days) as well as with frequent, time-consuming dressing changes (on average 9.6 times; every 1–3 days in PT burns)—also causing pain and anxiety—and a substantial need for grafting (i.e., in 21.5% of the patients). Furthermore, its known side effects include allergic reactions, argyria and neutropenia [54], and it also causes the wound bed’s discolouration, which can render wound evaluation and depth determination difficult [55].

Compared to SSD, collagenase combined with PMB showed no difference in TTRE in PT burns, whereas Silvasorb led to improved healing times in II/A and MD burns with 12.4 days (though TBSA% was not reported in Silvasorb-treated patients) [25]. Collagenase + PMB treatment markedly reduced the infection rates (to 2%) in the burned children, but it was associated with a high grafting rate (32%) and prolonged LOS (11.3 days). In the case of both Silvasorb and collagenase + PMB treatments, the dressing change rate was exceptionally high (13.5 and 11 times, respectively), which raises concerns about the recommendation of these treatments in paediatric PT burns.

Among the modern biosynthetic dressings, Biobrane and Transcyte had excellent efficacy with TTRE in PT burns of 10.63 days and T%/T of 0.63% for Biobrane, and TTRE 7.50 days and T%/T of 0.66% for Transcyte. In contrast, EzDerm was less efficient (TTRE in PT burns: 18.75 days; 0.23 T%/T) than SSD. The rates of infection and grafting were high in the case of EZDerm (37.0 and 23.9%, respectively) and Biobrane (12.9% for both), whereas Transcyte had a low rate of 5.0% for both. The need for reapplication was considerably low in the case of all three biosynthetic dressings, as shown by the small number of dressing changes in the case of EZDerm (*n* = 5), Biobrane (*n* = 3.4) and Transcyte (*n* = 1.5). Based on these results, biosynthetic treatments in children with PT burns are promising interventions, but in order to reduce the susceptibility to infection, and potentially the need for grafting, it is suggested that they should be applied in a combination with antimicrobial agents.

Silver foam dressings were mostly studied in small burns (<5 TBSA%) [43,44,45,46,47,48,49,50,51], though the wound’s area [17] and closure time [47] were not reported in the RCTs in the case of Aquacel products. By the 10th day of the treatment, the reepithelialisation was remarkably high in the case of Acticoat (93%) and Aquacel Ag (94%). Accordingly, the TTRE and T%/T in PT burns were reasonable in the case of Acticoat (14.2 days and 0.23%, respectively) and Mepilex (10.3 days and 0.28%, respectively). The number of dressing changes, infection rates and grafting needs were relatively low in the case of Acticoat (*n* = 2.7, 3.5% and 20.9%, respectively) and Mepilex Ag (*n* = 4.0, 16.4% and 3.3%, respectively). The LOS in the hospital was notably short (only 3.1 days) in children treated with Mepilex Ag. Aquacel Ag was also associated with a small need for dressing changes (*n* = 1.0) and low susceptibility to infections (2.3%). These results suggest that the silver foam dressings are efficient interventions in PT burns of children. However, before they can be firmly recommended for general practice, further studies are warranted to test their effect on more extensive burns as well.

Similarly to silver foam dressings, the combination therapies were mainly analysed on smaller burns, which could contribute to their favourable TTRE in PT burns, viz., 15.0 days for Biobrane + Recell, 10.6 days for Acticoat + Mepitel and 8.7 days for NPWT + Acticoat + Mepitel, as well as to the low T%/T values, which were 0.35% for Biobrane + Recell, 0.14% for Acticoat + Mepitel and 0.17% for NPWT + Acticoat + Mepitel. As an exception, the treatment with the combination of Biobrane and Acticoat resulted in a longer TTRE of 21 days and a higher T%/T of 0.87%. By the 10th day of the treatment, the reepithelialisation percentage was modest in the case of Acticoat + Mepitel (42.5%), and NPWT + Acticoat + Mepitel (68.6%), whereas it was remarkably high (95%) in children treated with Biobrane + Recell. Every intervention performed better than SSD in terms of the lower need for dressing changes, which were on average 6.9 for Biobrane + Acticoat, 4.8 for Biobrane + Recell, 3 for Acticoat + Mepitel and 2.4 for NPWT + Acticoat + Mepitel. It should be noted that the price of these treatments was higher than the cost of SSD, but the nursing and operating theatre time, along with the anaesthetic use and total cost, were high in the SSD group as well. The higher initial cost of the combination treatments is one of the main obstacles that prevent them from widespread use as burn therapies. Compared to SSD, the grafting rates were also reduced for Biobrane + Acticoat (13.9%), Acticoat + Mepitel (6.8%), NPWT + Acticoat + Mepitel (2.1%) and Biobrane + Recell (0%), but it should be mentioned that low grafting rates were also found with the sole treatment of Biobrane (6.2%), Transcyte (5%) and Mepilex Ag (3.3%). The infection rates were exceptionally high in the case of Biobrane + Acticoat (60%) and Biobrane + Recell (40%), which suggests that these combinations suppress the antimicrobial efficacy, while no infections were reported in children treated with Acticoat + Mepitel, which may indicate a powerful antimicrobial effect.

Radiation-sterilised AM allografts combined with antimicrobial agents [20,40,41] and tilapia xenografts [16] seem to be a surprisingly effective, low-cost solution, but their procurement and storage may be challenging. Their application seems comfortable and less painful during and in-between dressing changes, and they were also associated with the least number of average dressing changes: Tilapia (*n* = 3.0), AM (*n* = 1.3) and AM + Nystatin + PMB (*n* = 0.5). Moreover, the times needed for wound closure were among the lowest reported values as indicated by TTRE and T%/T in paediatric PT burns for Tilapia (10.1 days and 1.11%), AM (13.3 days and 0.56%) and AM + Nystatin + PMB (6.0 days and 2.00%). The infection rate in the case of AM + Nystatin + PMB was also very low (1.9%).

We collected several additional interventions that could reduce the time for reepithelialisation (as indicated by TTRE red%, see Methods for details) when they were supplemented to the treatment. The list of these interventions (with the corresponding burn severity and TTRE red%) included NPWT (II/A:12.6%; II/B: 14.3%), rhEGF (II/A: 20.2%; II/B: 20.7%), bFGF (II: 21.1%), vitamin E + C + Zinc (II: 23.7%), rhGM-CSF (II/B: 27.9%), wIRA (II/A: 30.8%) and heparin (II: 40.0%). While in several cases the cost of these interventions presents a considerable obstacle to their use, supplementation with vitamins, minerals and heparin can be promising and inexpensive adjuvants in burn therapies. It must be noted, however, that we could identify only a single report for each treatment, which warrants further research to establish the true efficacy of these additional interventions. For additional information on the reported advantages and drawbacks of each analysed treatment option see Text S1.

While a similar meta-analysis was previously conducted about the management of partial-thickness burn wounds in children in 2014 by Rashaan ZM et al., they could only compare the effects of non-silver treatment related to silver sulfadiazine due to the scarcity of articles [33]. In recent years, several new research studies were published on this topic; thus, we were able to analyse subgroups such as biosynthetic or silver foam dressings as well. Another systematic review was published by Vloemans et al. in 2014 called the Optimal treatment of partial-thickness burns in children [56]. They separated their findings based on evidence level (RCTs, cohort studies, case reports), but they were not able to conduct statistical analysis for similar reasons as Rashaan et al. Since then, more than twice as many RCTs have been published on this subject, new interventions have been tested—such as the combination or additional therapies—and more articles have been issued for the already existing therapies, which have now been added to this updated summary.

Limitations of our study must also be discussed. We aimed at collecting and evaluating articles strictly with the highest evidence level, namely RCTs; therefore, we had to exclude a lot of potentially relevant observational and case studies. It was surprising that despite the thorough review of the databases, we could identify a relatively low number of articles that fulfilled our inclusion criteria, especially if we consider the vast number of available treatment options. These RCTs were often describing a small and significantly heterogeneous population—due to the burns’ mixed sub-depth ratios and various average areas. The low number of studies limited our options for a more extensive meta-analysis on individual interventions and resulted predominantly in a qualitative synthesis of the available data. As another limitation of our study, it can also be mentioned that secondary outcomes were scarcely reported, and even then, they were assessed in diverse ways, mainly in the cases of cost, pain sensation and scar formation; hence, we were not able to compare these three endpoints. Moreover, some of the research was conducted over two decades ago when many of the more accurate diagnostic devices for burn depth classification and area determination (such as Laser Doppler Imaging) were not as widely available as now [57]. Thus, the preciseness of the older measurements might be questionable, and they were often unverifiable without photo documentation.

The assessed risk of bias was also high in general, largely resulting from the lack of reporting randomisation and blinding as well as the absence of (pre)trial protocols. Furthermore, most of the studies did not follow the CONSORT criteria—which may be one of the reasons behind the cause of missing data—or disclose funding sources and conflict of interests. In those cases when the founders were mentioned, they were usually the manufacturers of the evaluated intervention, which poses further risks for bias.

## 5. Conclusions

There are still many pieces missing from the grand picture of paediatric partial-thickness burn therapies; this review’s main goal was to summarise our current knowledge on the topic. Although the results presented in this article will most probably change over time, we aimed at highlighting currently unclear areas in our understanding and at facilitating further clinical studies in the field. A future network meta-analysis would provide sufficient information to differentiate between the efficacy of individual interventions, but a lot more RCTs are needed before we will be able to properly compare them.

Our primary recommendation for investigators is that superficial and deep second-degree burns in children should be analysed separately due to their significantly different characteristics. Furthermore, researchers should follow the CONSORT criteria and report predetermined outcomes of general interest (e.g., TBSA%, TTRE, T%/T, infection and grafting rates, number of dressing changes and LOS) along with their unique observations. Establishing a single, internationally accepted standard for pain and scar evaluation in paediatric burns would greatly advance this process. Another interesting future aspect could be the analysis of optimal dressing change rates.

While every intervention could facilitate the healing of second-degree paediatric burn wounds, individual data analysis showed remarkable differences in secondary outcomes that could not have been statistically proven because of the aforementioned limitations. When choosing the preferred intervention in paediatric PT burns, physicians should consider treatments with little need for dressing changes because these options require the lowest number of anaesthesias, as well as cause the least pain and discomfort for the children. Moreover, by reducing the reapplication rate, the operating theatres’ availability can be increased, and time can be saved for the healthcare providers, the advantages of which may result in a decrease in overall costs.

## Figures and Tables

**Table 1:** Characteristics of included studies.

Publication Data			Intervention	Demography		Aetiology			Burn Depth	Age (Years)			
Author	Year of Publication	Country		No. of Patients	Female (%)	Scald (%)	Contact (%)	Flame (%)		Mean	SD	Min (Months)	Max
**Barbosa et al. [53]**	2009	Brazil	**Vitamin C&E + Zn**	17	35.3	NR	NR	NR	II	4.51	4.32	NR	NR
			**placebo**	15	33.3	NR	NR	NR		4.53	3.74	NR	NR
**Barret et al. [18]**	2000	USA, Texas	**Biobrane**	10	30	80	0	20	II	3.1	0.5′	NR	17
			**SSD (Silvadene)**	10	20	70	0	30		3.7	0.6′	NR	17
**Branski et al. [40]**	2007	USA, Texas	**Nystatin** **+ PMB**	49	28.57	45	0	55	II	7	4	NR	NR
			**AM + Nystatin + PMB**	53	30.19	43	0	57		7	4	NR	NR
**Brown et al. [47]**	2016	New Zealand	**Acticoat**	41	46.67	91	9	0	II	4.3	4	NR	15
			**Aquacel Ag foam**	40	43.18	95	5	0		3	3.5	NR	15
**Bugmann et al. [24]**	1998	Switzerland	**Mepitel**	36	46.34	68.3	26.8	4.87	II	3.29	3.09	3	15
			**SSD (Flamazin)**	30	42.86	60	25.7	11.43		3.43	3.7	3	15
**Caruso et al. [17]**	2006	USA, Arizona	**Aquacel Ag foam**	13	NR	NR	NR	NR	II/A + MD	NR	NR	2	16
			**SSD**	19	NR	NR	NR	NR		NR	NR	2	16
**Cassidy et al. [42]**	2005	USA, Kansas	**Duoderm**	37	NR	NR	NR	NR	II/A + MD	NR	NR	36	18
			**Biobrane**	35	NR	NR	NR	NR		NR	NR	36	18
**Frear et al. [45]**	2020	Australia	**Acticoat + Mepitel**	54	42.59	65	33	2	II	4 *	NR	12 **	9^
			**NPWT+ Acticoat +** **Mepitel**	47	59.57	60	36	4		4 *	NR	12 **	8^
**Gee Kee et al. [46]**	2015	Australia	**Acticoat**	31	41.94	58.1	35.5	3.2	II	1	NR	1	5
			**Acticoat** **+ Mepitel**	32	34.38	62.5	34.4	3.1		1	NR	1	4
			**Mepilex**	33	51.52	54.5	42.4	0		1	NR	1	4
**Glat et al. [25]**	2009	USA, Pennsylvania	**Silvasorb**	12	NR	NR	NR	NR	II/A + MD	3.58	2.43	13	5
			**SSD (Silvadene)**	12	NR	NR	NR	NR		1.9	1.13	9	9
**Gotschall et al. [23]**	1998	USA, Wa-shington	**Mepitel**	33	NR	100	0	0	II	NR	NR	NR	12
			**SSD**	30	NR	100	0	0		NR	NR	NR	12
**Hartel et al. [36]**	2007	Germany	**wIRA(75%)** **+ VIS**	10	NR	NR	NR	NR	II/A	NR	NR	NR	NR
			**VIS (placebo)**	10	NR	NR	NR	NR		NR	NR	NR	NR
**Hayashida et al. [37]**	2012	Japan	**bFGF**	15	NR	66.7	13.3	20	II	NR	NR	8	2.67
			**placebo (Ekzalb)**	15	NR	73.3	6.7	20		NR	NR	8	2.67
**Healy et al. [44]**	1989	UK	**EZDerm**	9	NR	NR	NR	NR		2.6	0.6′	NR	NR
**Hyland et al. [49]**	2018	Australia	**Biobrane + Acticoat**	10	30	NR	NR	NR	MD	NR	NR	0	16
			**Acticoat**	10	20	NR	NR	NR		NR	NR	0	16
**Jiaao et al. [34]**	2010	China	**rhGM-CSF**	15	NR	NR	NR	NR	II/B	5.3	NR	NR	NR
			**placebo (hydrogel matrix)**	15	NR	NR	NR	NR		5.3	NR	NR	NR
**Karlsson et al. [48]**	2019	Sweden	**Ezderm**	30	36.67	100	0	0	II	1.75 *	NR	11 **	4.92 ^
			**Mepilex**	28	42.86	100	0	0		1.42 *	NR	8 **	2.92 ^
**Kumar et al. [21]**	2004	Australia	**Biobrane**	17	NR	NR	NR	NR	II	3.6	NR	NR	NR
			**Transcyte**	20	NR	NR	NR	NR		3.6	NR	NR	NR
			**SSD (Silvazin)**	21	NR	NR	NR	NR		3.6	NR	NR	NR
**Lal et al. [22]**	2000	USA, Texas	**Biobrane**	34	44.12	100	0	0	II/A	2.8	0.5′	0	17
			**SSD**	45	33.33	100	0	0		3.4	0.6′	0	17
**Liang et al. [39]**	2007	China	**rhEGF**	30	NR	NR	NR	NR	II/A	NR	NR	NR	14
				30	NR	NR	NR	NR	II/B	NR	NR	NR	14
			**placebo (saline gauze)**	30	NR	NR	NR	NR	II/A	NR	NR	NR	14
				30	NR	NR	NR	NR	II/B	NR	NR	NR	14
**Lima Júnior et al. [16]**	2019	Brazil	**Tilapia**	15	33.3	93.3	0	6.67	II/A	5.67	3.66	24	12
			**SSD**	15	46.67	80	0	20		5.2	2.7	24	12
**Mostaque et al. [20]**	2011	Bangladesh	**AM**	51	52.9	82.4	0	17.6	II/A	3.61	2.31	0.03	12
				22	NR	NR	NR	NR	II/B	NR	NR	0.03	12
			**SSD**	51	51	49	0	51	II/A	4.03	2.4	0.03	12
				36	NR	NR	NR	NR	II/B	NR	NR	0.03	12
**Muganza et al. [26]**	2014	South Africa	**Biobrane** **+ Acticoat**	26	46.15	NR	NR	NR	II	2.3 *	NR	20.4 **	4.1 ^
			**SSD**	19	57.89	NR	NR	NR		2.7 *	NR	19.2 **	4.1 ^
**Omranifard et al. [41]**	2011	Iran	**AM**	34	29.41	NR	NR	NR	II/B	5.4	7.5	NR	18
			**autograft**	32	34.38	NR	NR	NR		4.4	6.9	NR	18
**Ostlie et al. [19]**	2012	USA, Kansas	**Collagenase** **+ PMB**	50	42	NR	NR	NR	II	4.8	4.5	2	18
			**SSD**	50	30	NR	NR	NR		5.1	4.5	2	18
**Venkatachalapathy et al. [38]**	2012	India	**Heparin**	50	NR	NR	NR	NR	II	NR	NR	NR	NR
			**Sulphur-based cream**	50	NR	NR	NR	NR		NR	NR	NR	NR
**Shen et al. [35]**	2013	China	**NPWT**	145	NR	100	0	0	II/A	NR	NR	NR	NR
**Wood et al. [43]**	2012	Australia	**Biobrane**	4	50	100	0	0	II	4.95	3.91	8	9
			**Biobrane** **+ ReCell**	5	40	100	0	0		1.32	0.55	8	9
**Zheng et al**. [27]	2019	China	**NPWT**	32	43.75	NR	NR	NR	II/B	3.9	1.6	NR	NR
			**SSD**	32	37.5	NR	NR	NR		3.8	1.7	NR	NR

Different markings were used when the analysed endpoints were given in * = median, ** = IQR25, ^ = IQR75 and ’ = SEM. (II = Partial-thickness burn injury (PT); II/A = superficial PT; II/B = deep PT; AM= amnion membrane; MD = mid-dermal or mixed-depth burn injury; NPWT = negative pressure wound therapy; NR = not reported; PMB = polymyxin b; SD = standard deviation; SSD = silver sulphadiazine; TBSA% = burned area of the total body surface; VIS = visible spectrum light; wIRA = water-filtered infrared.)

**Table 2:** Average wound closure time in paediatric PT burns. The synopsis of the available interventions’ reported healing potential with an unknown sub-depth ratio.

Depth: II	Publication Data	No. of Patients (*n*)	TBSA(%)		TTRE (Days)		TBSA%/TTRE	TTRE Red%
Intervention	Author (Year of Publication)		Mean	SD	Mean	SD		
**Acticoat + Mepitel SUM**		**86**	**1.42**		**10.61**		**0.14**	
Acticoat + Mepitel	Frear et al. (2020) [45]	54	* 1.35	0.76	* 10.7	4.57	0.13	
Acticoat + Mepitel	Gee Kee et al. (2015) [46]	32	* 1.53	1.94	* 10.35	3.91	0.15	
**NPWT + Acticoat** **+ Mepitel**	Frear et al. (2020) [45]	47	* 1.5	0.76	* 8.71	3.06	**0.17**	
**EZDerm SUM**		**39**	**4.26**		**18.75**		**0.23**	
EZDerm	Healy et al. (1989) [44]	9	1.8	3.75	12.9	4.2	0.14	
EZDerm	Karlsson et al. (2019) [48]	30	** 5	NR	20.5	NR	0.24	
**Acticoat SUM**		**41**	**3.23**		**14.18**		**0.23**	
Acticoat	Hyland et al. (2018) [49]	10	** 8.5	NR	26.5	NR	0.32	
Acticoat	Gee Kee et al. (2015) [46]	31	1.53	1.94	10.21	5.47	0.15	
**Mepilex SUM**		**61**	**2.85**		**10.29**		**0.28**	
Mepilex	Karlsson et al. (2019) [48]	28	** 5	NR	** 15	NR	0.33	
Mepilex	Gee Kee et al. (2015) [46]	33	* 1.03	1.16	6.29	3.1	0.16	
**Biobrane + ReCell**	Wood et al. (2012) [43]	**5**	**5.2**	3.19	**15**	3.54	**0.35**	
**SSD SUM**		**110**	**7.21**		**18.29**		**0.39**	
SSD	Gotschall et al. (1998) [23]	30	5.1	2.2	27.6	NR	0.19	
SSD	Muganza et al. (2014) [26]	19	21	7.1	23.7	9.4	0.89	
SSD	Barret et al. (2000) [18]	10	7.8	2.85	16.1	2.21	0.48	
SSD	Bugmann et al. (1998) [24]	30	1.92	2.05	11.26	6.02	0.17	
SSD	Kumar et al. (2004) [21]	21	5	NR	11.2	NR	0.45	
**placebo (Ekzalb)**	Hayashida et al. (2012) [37]	**15**	**8.3**	2.9	**17.5**	3.1	**0.47**	
**bFGF**	Hayashida et al. (2012) [37]	**15**	**7**	2.6	**13.8**	2.4	**0.51**	**21.14**
**Mepitel SUM**		**69**	**4.97**		**8.98**		**0.55**	
Mepitel	Gotschall et al. (1998) [23]	33	6.8	3.4	10.5	NR	0.65	
Mepitel	Bugmann et al. (1998) [24]	36	3.29	3.09	7.58	3.12	0.43	
**Biobrane SUM**		**31**	**6.65**		10.63		**0.63**	
Biobrane	Wood et al. (2012) [43]	4	8	5.23	17.75	4.99	0.45	
Biobrane	Barret et al. (2000) [18]	10	8.9	15.5	9.7	2.21	0.92	
Biobrane	Kumar et al. (2004) [21]	17	5	NR	9.5	NR	0.53	
**Transcyte**	Kumar et al. (2004) [21]	**20**	5	NR	7.5	NR	**0.66**	
**Biobrane +** **Acticoat SUM**		**36**	**18.11**		**20.95**		**0.87**	
Biobrane + Acticoat	Muganza et al. (2014) [26]	26	22	7.5	21.7	9	1.01	
Biobrane + Acticoat	Hyland et al. (2018) [49]	10	** 8	NR	19	NR	0.42	
**Nystatin + PMB**	Branski et al. (2007) [40]	**49**	11	6	**8**	2	**1.38**	
**AM** **+ Nystatin +PMB**	Branski et al. (2007) [40]	**53**	12	7	**6**	2	**2**	
**Vitamin C&E + Zinc + TT**	Barbosa et al. (2009) [53]	**15**	16.2	5.3	**7.5**	NR	**2.16**	**23.67**

Numbers marked with a single star (*) were converted from median to mean. Two stars (**) signify that the number could not be converted from median due to missing IQR75 or reporting the range in 10 and 90 percentiles (II = partial-thickness burn injury (PT); AM = amnion membrane; NPWT = negative pressure wound therapy; NR = not reported; PMB = polymyxin b; SSD = silver sulphadiazine; SUM = the summarised values of the same interventions; TBSA% = burned area of the total body surface; TT = traditional treatment; TTRE = time to reepithelialisation; TTRE red% = the percentage of time reduction, with the addition of the intervention; TBSA%/TTRE = the area of regeneration per day).

**Table 3:** Average grafting ratios. Comparison of the need for surgical intervention from the RCTs about PT burns in children, measured in percentages.

Intervention and Burn Depth	Publication Data	No. of Patients (*n*)	Grafted	
	Author (Year of Publication)		No. (*n*)	%
**placebo (VIS) II/A**	Hartel et al. (2007) [36]	**24**	14	**58.33**
**wIRA(75%) + VIS II/A**	Hartel et al. (2007) [36]	**21**	11	**52.38**
**placebo (Ekzalb) II**	Hayashida et al. (2012) [37]	**15**	5	**33.33**
**bFGF II**	Hayashida et al. (2012) [37]	**15**	5	**33.33**
**Collagenase + PMB II**	Ostlie et al. (2012) [19]	**50**	16	**32**
**EZDerm II**		**46**	11	**23.91**
EZDerm	Healy et al. (1989) [44]	16	7	43.75
EZDerm	Karlsson et al. (2019) [48]	30	4	13.33
**SSD II**		**180**	29	**21.48**
**Acticoat II**		**43**	9	**20.93**
Acticoat	Hyland et al. (2018) [49]	10	7	70
Acticoat	Gee Kee et al. (2015) [46]	33	2	6.06
**Nystatin + PMB II**	Branski et al. (2007) [40]	**59**	10	**16.95**
**SSD II + II/A**		**135**	29	**16.1**
SSD	Ostlie et al. (2012) [19]	50	18	36
SSD	Kumar et al. (2004) [21]	21	5	23.81
SSD	Barret et al. (2000) [18]	10	0	0
SSD	Bugmann et al. (1998) [24]	35	5	14.28
SSD	Muganza et al. (2014) [26]	19	1	5.26
**SSD II/A**	Lal et al. (2000) [22]	45	0	**0**
**Biobrane + Acticoat II**		**36**	5	**13.89**
**Biobrane + Acticoat**	Hyland et al. (2018) [49]	10	4	40
**Biobrane + Acticoat**	Muganza et al. (2014) [26]	26	1	3.85
**AM + Nystatin + PMB II**	Branski et al. (2007) [40]	**61**	8	**13.11**
**Biobrane II**		**31**	4	**12.9**
**Mepitel II**	Bugmann et al. (1998) [24]	**41**	5	**12.19**
**Acticoat + Mepitel II**		**88**	6	**6.82**
Acticoat + Mepitel	Frear et al. (2020) [45]	54	4	7.41
Acticoat + Mepitel	Gee Kee et al. (2015) [46]	34	2	5.89
**Biobrane II + II/A**		**65**	4	**6.15**
Biobrane	Wood et al. (2012) [43]	4	1	25
Biobrane	Kumar et al. (2004) [21]	17	3	17.65
Biobrane	Barret et al. (2000) [18]	10	0	0
**Biobrane II/A**	Lal et al. (2000) [22]	34	0	**0**
**Transcyte II**	Kumar et al. (2004) [21]	**20**	1	**5**
**Mepilex II**		**61**	2	**3.28**
Mepilex	Karlsson et al. (2019) [48]	28	2	7.14
Mepilex	Gee Kee et al. (2015) [46]	33	0	0
**NPWT + Acticoat + Mepitel II**	Frear et al. (2020) [45]	**47**	1	**2.13**
**Biobrane + ReCell II**	Wood et al. (2012) [43]	**5**	0	**0**

(II = Partial-thickness burn injury (PT); II/A = superficial PT; AM = amnion membrane; NPWT = negative pressure wound therapy; PMB = polymyxin b; RCTs = randomised controlled trials; SSD = silver sulphadiazine; VIS = visible spectrum light; wIRA = water-filtered infrared A.)

**Table 4:** Mean frequency of dressing changes. A brief about the RCTs’ average reported need for dressing reapplication in the PT management of children.

Intervention and Burn Depth	Publication Data	No. of Patients	Dressing Changes	
	Author (Year of Publication)		Mean	SD
**SSD II + II/A**		**198**	**25.16**	
**Silvasorb II/A+ MD**	Glat et al. (2009) [25]	**12**	**13.5**	4.7
**Collagenase + PMB II**	Ostlie et al. (2012) [19]	**50**	**11**	4.1
**SSD II ex. Mostaque**		**132**	**9.56**	
SSD	Mostaque et al. (2011) [20]	51	65.53	18.23
SSD	Glat et al. (2009) [25]	12	13.42	8.26
SSD	Ostlie et al. (2012) [19]	50	11	3.8
SSD	Muganza et al. (2014) [26]	19	10.7	3.8
SSD	Kumar et al. (2004) [21]	21	9.2	NR
SSD	Bugmann et al. (1998) [24]	30	5.13	2.9
**SSD II/A**	Lima Júnior et al. (2019) [16]	**15**	**9.27**	1.39
**Biobrane + Acticoat II**		**36**	**6.87**	
Biobrane + Acticoat	Muganza et al. (2014) [26]	26	7.6	4.8
Biobrane + Acticoat	Hyland et al. (2018) [49]	10	* 5	NR
**Nystatin + PMB II**	Branski et al. (2007) [40]	**49**	**6**	3
**EZDerm II**	Karlsson et al. (2019) [48]	**30**	* 5	NR
**Biobrane +** **ReCell II**	Wood et al. (2012) [43]	**5**	**4.8**	1.3
**Mepilex II**	Karlsson et al. (2019) [48]	**28**	* 4	NR
**Mepitel II**	Bugmann et al. (1998) [24]	**36**	**3.64**	1.5
**Biobrane II**		**21**	**3.37**	
Biobrane	Wood et al. (2012) [43]	4	7.5	2.64
Biobrane	Kumar et al. (2004) [21]	17	2.4	NR
**Acticoat + Mepitel II**	Frear et al. (2020) [45]	**54**	**3**	1.48
**Tilapia II/A**	Lima Júnior et al. (2019) [16]	**15**	**3**	0.76
**Acticoat II**		**51**	**2.69**	
Acticoat	Hyland et al. (2018) [49]	10	* 5.5	NR
Acticoat	Brown et al. (2016) [47]	41	2	0.2
**NPWT + Acticoat +** **Mepitel II**	Frear et al. (2020) [45]	**47**	**2.43**	0.86
**NPWT II/A**	Shen et al. (2013) [35]	**145**	**2.05**	0.22
**Transcyte II**	Kumar et al. (2004) [21]	**20**	**1.5**	NR
**AM II**	Mostaque et al. (2011) [20]	**51**	**1.33**	0.55
**Aquacel Ag II**	Brown et al. (2016) [47]	**40**	**1**	0.1
**AM + Nystatin +** **PMB II**	Branski et al. (2007) [40]	**53**	**0.5**	2

Numbers marked with a single star (*) were converted from median to mean. (II = Partial-thickness burn injury (PT); II/A = superficial PT; AM = amnion membrane; ex. Mostaque = this study was excluded from a part of the analysis due to its open treatment regime; MD = mid-dermal or mixed-depth burn injury; NPWT = negative pressure wound therapy; NR = not reported; PMB= polymyxin b; RCTs = randomised controlled trials; SD = standard deviation; SSD = silver sulphadiazine.)

**Table 5:** Mean infection chance. Summary of percentages when infection occurred during the treatment of PT in children.

Intervention and Burn Depth	Publication Data	No. of Patients (*n*)	Infected	
	Author (Year of Publication)		No. (*n*)	%
**Biobrane + Acticoat II**	Hyland et al. (2018) [49]	**10**	6	**60**
**Biobrane + ReCell II**	Wood et al. (2012) [43]	**5**	2	**40**
**EZDerm II**		**46**	17	**36.96**
EZDerm	Healy et al. (1989) [44]	16	8	50
EZDerm	Karlsson et al. (2019) [48]	30	9	30
**NPWT II/A**	Shen et al. (2013) [35]	**145**	39	**26.9**
**Mepilex II**		**61**	10	**16.39**
Mepilex	Karlsson et al. (2019) [48]	28	10	35.72
Mepilex	Gee Kee et al. (2015) [46]	33	0	0
**Biobrane II**		**31**	4	**12.9**
**SSD II**		**141**	13	**9.22**
**Biobrane II + II/A**		**65**	5	**7.69**
Biobrane	Wood et al. (2012) [43]	4	1	25
Biobrane	Kumar et al. (2004) [21]	17	3	17
Biobrane	Barret et al. (2000) [18]	10	0	0
**Biobrane II/A**	Lal et al. (2000) [22]	34	1	**2.9**
**SSD II + II/A**		**186**	14	**7.53**
SSD	Kumar et al. (2004) [21]	21	5	24
SSD	Ostlie et al. (2012) [19]	50	7	14
SSD	Bugmann et al. (1998) [24]	30	1	3.33
SSD	Gotschall et al. (1998) [23]	30	0	0
SSD	Barret et al. (2000) [18]	10	0	0
**SSD II/A**	Lal et al. (2000) [22]	**45**	1	**2.2**
**Transcyte II**	Kumar et al. (2004) [21]	**20**	1	**5**
**Mepitel II**		**72**	3	**4.17**
Mepitel	Gotschall et al. (1998) [23]	36	3	8.3
Mepitel	Bugmann et al. (1998) [24]	36	0	0
**Nystatin + PMB II**	Branski et al. (2007) [40]	**49**	2	**4.08**
**Acticoat II**		**86**	3	**3.49**
Acticoat	Brown et al. (2016) [47]	45	2	4.44
Acticoat	Hyland et al. (2018) [49]	10	1	10
Acticoat	Gee Kee et al. (2015) [46]	31	0	0
**Aquacel Ag II**	Brown et al. (2016) [47]	**44**	1	**2.27**
**Collagenase +** **PMB II**	Ostlie et al. (2012) [19]	**50**	1	**2**
**AM + Nystatin +** **PMB II**	Branski et al. (2007) [40]	**53**	1	**1.89**
**Acticoat +** **Mepitel II**	Gee Kee et al. (2015) [46]	**32**	0	**0**

(II = Partial-thickness burn injury (PT); II/A = superficial PT; AM = amnion membrane; NPWT = negative pressure wound therapy; PMB = polymyxin b; SD = standard deviation; SSD = silver sulphadiazine.)

## Data Availability

Data are presented in the manuscript and Supplementary files.

## Supplementary Materials

The following supporting information can be downloaded at: https://www.mdpi.com/article/10.3390/life12050619/s1, **Figure S1: Risk of bias assessment**. The RCTs compared paediatric patients with PT burns. **Figure S2: Pooled TTRE of the interventions, adjusted by burn depth**. The groups were II/A, II and II/B injuries in children, and the TTRE was measured in days. **Figure S3: Mean TTRE of the interventions, modified by burn area.** The categories were <5, 5–10, 10–25 TBSA% paediatric PT injuries, and the TTRE was calculated in days. **Figure S4: SSD’s mean TTRE stratified by depth.** The classifications were paediatric II/A and II burns and the TTRE was determined in days. **Figure S5: Average TTRE of SSD, modified by burn area**. The area cohorts were <10 and 10–25 TBSA% PT injuries, and the TTRE was defined in days. **Figure S6: The mean TTRE of SSD and Biobrane.** The meta-analysis of TTRE in children with PT was measured in days. **Figure S7: Average grafted percentages.** Treatment groups were paediatric PT patients with SSD, silver foam and biosynthetic dressings. **Figure S8: Mean non-grafted patient ratio with SSD and Biobrane.** The percentage comparison of successful conservative treatment in PT injuries of children. **Figure S9: Average infected population percentages.** Treatment comparison **of** SSD, biosynthetic and silver foam dressings as well as Biobrane only in paediatric PT injuries. **Figure S10: Mean non-infected patients’ ratio of SSD compared to Biobrane.** The meta-analysis compares the frequencies of complication-free paediatric PT burn treatments, in percentages. **Table S1: RoB2 analysis. Table S2: Mean TTRE of paediatric II/A treatments.** TTRE values were calculated in days. (II/A = Superficial partial-thickness injury; NA = not applicable; NPWT = negative pressure wound therapy, NR = not reported; SD = standard deviation; SSD = silver sulphadiazine; SUM = the summarized values of the same interventions; TBSA% = burned area of the total body surface; TT = traditional treatment; TTRE = time to reepithelialisation; TBSA%/TTRE= the area of regeneration per day; TTRE red% = the percentage of time reduction, with the addition of the intervention; VIS = visible spectrum light; wIRA = water-filtered infrared A.) **Table S3: The average TTRE in paediatric II/A and MD burns.** The TTRE was measured in days. No description was provided about the TBSA% in these instances, thus the T%/T ratios could not be calculated. (II/A = Superficial partial-thickness injury; MD = mid-dermal burn; NR = not reported; SD = standard deviation; SSD = silver sulphadiazine; SUM = the summarized values of the same interventions; TBSA% = burned area of the total body surface; TTRE = time to reepithelialisation.) **Table S4: Mean TTRE summary of II/B injuries in children.** The TTRE was calculated in days. (II/B = Deep partial-thickness burn injury; AM = amnion membrane; NA = not applicable; NPWT = negative pressure wound therapy; NR = not reported; SD = standard deviation; SSD = silver sulphadiazine; SUM = the summarised values of the same interventions; TBSA% = burned area of the total body surface; TTRE = time to reepithelialisation; TBSA%/TTRE = the area of regeneration per day.) **Table S5: Average percentage of reepithelialisation on the tenth day (day 10 RE%) in paediatric PT injuries.** (II = Partial-thickness burn injury (PT); II/A = superficial PT; AM = amnion membrane; day 10 RE = the fraction of reepithelialisation on the tenth day; NA = not applicable; NPWT = negative pressure wound therapy; NR = not reported; SSD = silver sulphadiazine; TBSA% = burned area of the total body surface; TTRE = time to reepithelialisation; TBSA%/TTRE = the area of regeneration per day.) **Table S6: Mean length of stay.** The time children spent (days) inside the hospital while being treated with different regimens for PT burns. (II = Partial-thickness burn injury (PT); II/A = superficial PT; AM = amnion membrane; LOS = length of hospital stay; NPWT = negative pressure wound therapy; NR = not reported; PMB = polymyxin b; SD = standard deviation; SSD = silver sulphadiazine.) **Text S1: Reviews of the analysed interventions. Text S2: Search keys**. References [16,17,18,19,20,21,22,23,24,25,26,27,34,35,36,37,38,39,40,41,42,43,44,45,46,47,48,49,50,51,53,58,59,60,61,62,63,64,65,66,67,68,69,70,71,72,73,74,75,76,77,78,79,80,81,82,83,84,85,86,87] are mentioned in the Supplementary Materials.
