# Supplementary material for: Paediatric Partial-Thickness Burn Therapy: A Meta-Analysis and Systematic Review of Randomised Controlled Trials
Source: Life (Basel). 2022 Apr 21;12(5):619. doi: 10.3390/life12050619 (PMC9144506; doi:10.3390/life12050619)
Supplement: Supplementary file 1 [file life-12-00619-s001.zip › STables.revised/S2Table.pdf]

**Depth: II/A**	**Publication data**	**No. of patients**	**TBSA%**		**TTRE (days)**		**TBSA/ TTRE**	**TTRE red%**
**Intervention**	**Author (year of publication)**		**mean**	**SD**	**mean**	**SD**		
**Biobrane**	**Lal et al. (2000)**	**34**	**11.5**	NR	**23.6**	2.9	**0.49**	
**SSD SUM**		**75**	**10.98**		**20.83**		**0.53**	
SSD	**Lal et al. (2000)**	**45**	11.8	NR	26.5	2	0.45	
SSD	**Mostaque et al. (2011)**	**15**	9.39	4.62	14.2	0.96	0.66	
SSD	**Edmar et al. (2019)**	**15**	10.13	4.16	10.47	0.74	0.97	
**Amnion**	**Mostaque et al. (2011)**	**29**	**7.39**	3.63	**13.33**	0.95	**0.56**	
**Tilapia**	**Edmar et al. (2019)**	**15**	**11.13**	4.94	**10.07**	0.46	**1.11**	
**NPWT**	**Shen et al. (2013)**	**145**	NR	NR	**9.2**	0.6	NA	**12.57**
**rhEGF**	**Liang et al. (2007)**	**30**	NR	NR	**8.83**	2.48	NA	**20.16**
**wIRA(75%)+VIS**	**Illing et al. (2008)**	**10**	NR	NR	**9**	NR	NA	**30.77**
**placebo (VIS)**	**Illing et al. (2008)**	**10**	NR	NR	**13**	NR	NA	NA
**placebo (saline gauze)**	**Liang et al. (2007)**	**30**	NR	NR	**11.06**	3.05	NA	NA
**TT**	**Shen et al. (2013)**	**162**	NR	NR	**10.1**	1.6	NA	NA
