# Supplementary material for: Paediatric Partial-Thickness Burn Therapy: A Meta-Analysis and Systematic Review of Randomised Controlled Trials
Source: Life (Basel). 2022 Apr 21;12(5):619. doi: 10.3390/life12050619 (PMC9144506; doi:10.3390/life12050619)
Supplement: Supplementary file 1 [file life-12-00619-s001.zip › STables.revised/S3Table.pdf]

Depth: II/A + MD	Publication data	No. of patients	TTRE (days)	
**Intervention**	**Author (year of publication)**		**mean**	**SD**
**SSD SUM**		**31**	**14.74**	
SSD	**Caruso et al. (2006)**	19	16	NR
SSD	**Glat et al. (2009)**	12	12.75	7.45
**Aquacel Ag**	**Caruso et al. (2006)**	**13**	**14**	NR
**Silvasorb**	**Glat et al. (2009)**	**12**	**12.42**	3.58
**Biobrane**	**Cassidy et al. (2005)**	**35**	**12.24**	5.1
**Duoderm**	**Cassidy et al. (2005)**	**37**	**11.21**	6.5
