# Supplementary material for: Paediatric Partial-Thickness Burn Therapy: A Meta-Analysis and Systematic Review of Randomised Controlled Trials
Source: Life (Basel). 2022 Apr 21;12(5):619. doi: 10.3390/life12050619 (PMC9144506; doi:10.3390/life12050619)
Supplement: Supplementary file 1 [file life-12-00619-s001.zip › STables.revised/S4Table.pdf]

Depth: II/B	Publication data	No. of patients	TBSA(%)		TTRE(days)		TBSA%/ TTRE	TTRE red%
Intervention	Author (year of publication)		mean	SD	mean	SD		
**AM**	**Mostaque et al. (2011)**	**22**	**2.25**	3.14	**21.59**	1.44	**0.1**	
**SSD SUM**		**68**	**5.48**		**25.69**		**0.21**	
SSD	**Zheng et al. (2019)**	**32**	5.8	1.6	27.9	1.8	0.21	
SSD	**Mostaque et al. (2011)**	**36**	5.2	5.45	23.72	1.5	0.22	
**NPWT**	**Zheng et al. (2019)**	**32**	**5.5**	2.2	**23.9**	2.3	**0.23**	**14.3**
**rhEGF**	**Liang et al. (2007)**	**30**	NR	NR	**15.12**	3.19	NA	**20.7**
**rhGM-CSF**	**Jiaao et al. (2010)**	**15**	NR	NR	**13.47**	1.08	NA	**27.9**
**placebo (saline gauze)**	**Liang et al. (2007)**	**30**	NR	NR	**19.06**	3.07		
**placebo (hydrogel matrix)**	**Jiaao et al. (2010)**	**15**	NR	NR	**18.69**	2.35		
