# Supplementary material for: Paediatric Partial-Thickness Burn Therapy: A Meta-Analysis and Systematic Review of Randomised Controlled Trials
Source: Life (Basel). 2022 Apr 21;12(5):619. doi: 10.3390/life12050619 (PMC9144506; doi:10.3390/life12050619)
Supplement: Supplementary file 1 [file life-12-00619-s001.zip › STables.revised/S5Table.pdf]

Intervention	Burn depth	Publication data	No. of patients	TBSA(%)	TTRE(days)	day 10 RE(%)	TBSA%/ TTRE
		Author (year of publication)		mean	mean		
SSD	II/A	Edmar et al. (2019)	15	10.13	10.47	53.33	0.97
Tilapia		Edmar et al. (2019)	15	11.13	10.07	86.67	1.11
Acticoat+Mepitel	II	Frear et al. (2020)	54	1.35	10.7	42.45	0.13
SSD		Bugmann et al. (1998)	30	1.92	11.26	53.33	0.17
NPWT+Acticoat+Mepitel		Frear et al. (2020)	44	1.5	8.71	68.57	0.17
Mepitel		Bugmann et al. (1998)	36	3.29	7.58	77.77	0.44
Biobrane		Wood et al. (2012)	4	8.00	17.75	83.20	0.63
Acticoat		Brown et al. (2016)	41	2.40	NR	93	NA
Aquacel Ag		Brown et al. (2016)	40	2.50	NR	94	NA
Biobrane + ReCell		Wood et al. (2012)	5	5.20	15	95	0.35
