# Supplementary material for: Paediatric Partial-Thickness Burn Therapy: A Meta-Analysis and Systematic Review of Randomised Controlled Trials
Source: Life (Basel). 2022 Apr 21;12(5):619. doi: 10.3390/life12050619 (PMC9144506; doi:10.3390/life12050619)
Supplement: Supplementary file 1 [file life-12-00619-s001.zip › STables.revised/S6Table.pdf]

Intervention	Publication data	No. of patients	LOS (days)	
	Author (year of publication)		mean	SD
**Biobrane+Acticoat**	**Muganza et al. (2014)**	**26**	**27.77**	25.93
**Autograft**	**Omranifard et al. (2011)**	**32**	**20.2**	10.3
**SSD II**		**130**	**13.77**	
**AM II**		**85**	**11.37**	
AM	**Omranifard et al. (2011)**	34	12.4	7.6
AM	**Mostaque et al. (2011)**	**51**	10.69	3.87
**Collagenase+PMB**	**Ostlie et al. (2012)**	**50**	**11.3**	5.8
**SSD II+II/A**		**175**	**10.46**	
SSD	**Muganza et al. (2014)**	19	26.83	21.2
SSD	**Mostaque et al. (2011)**	51	13.43	5.13
SSD	**Ostlie et al. (2012)**	50	11.2	5.2
SSD	**Barret et al. (2000)**	10	3.6	NR
**SSD II/A**	**Lal et al. (2000)**	45	**5.79**	NR
**EZDerm**	**Karlsson et al. (2019)**	**30**	**3.36**	3.94
**Mepilex**	**Karlsson et al. (2019)**	**28**	**3.12**	2.2
**Biobrane II+II/A**		**44**	**2.36**	
**Biobrane**	**Barret et al. (2000)**	10	**1.5**	NR
**Biobrane II/A**	**Lal et al. (2000)**	34	**2.62**	NR
**AM+Nystatin+PMB**	**Branski et al. (2007)**	**53**	**2**	3
**Nystatin+PMB**	**Branski et al. (2007)**	**49**	**2**	3
