# Supplementary material for: Paediatric Partial-Thickness Burn Therapy: A Meta-Analysis and Systematic Review of Randomised Controlled Trials
Source: Life (Basel). 2022 Apr 21;12(5):619. doi: 10.3390/life12050619 (PMC9144506; doi:10.3390/life12050619)
Supplement: Supplementary file 1 [file life-12-00619-s001.zip › STexts/S1 Text.v5.pdf]

S1 Text: Reviews of the analysed interventions.

Silver sulphadiazine (SSD) [1–12] contains a unique sulphonamide component that does not inhibit folic acid synthesis, suggesting that the silver ion content mainly causes its antibacterial qualities. The ionic silver released from SSD only has limited eschar penetration; thus, they act superficially. SSD's precise bactericidal mechanism of action is still unknown. However, it may damage DNA replication and increase cell wall permeability through the direct alteration of the membrane and via the production of free radicals. Wounds can be treated while open (SSD only) or closed with the addition of gauze dressings [13].

DuoDERM® [14,15] (ConvaTec Group PLC, Reading, England) is an opaque, biodegradable, and breathable occlusive hydrocolloid dressing that can be worn for up to seven days. It has an outer film that provides an impermeable barrier and a foam layer designed for thermal and mechanical protection. Additionally, it contains an inner honeycomb matrix of hydrocolloid compounds that absorb exudate to form a soft, moist gel [16].

Mepitel® [8,9,17–20] (Mölnlycke Health Care, Gothenburg, Sweden) is a semi-transparent, low-adherent, porous wound contact layer that comprises an elastic polyamide net covered with silicone and contains no biological compounds. The silicone coating is slightly adhesive, which helps it to be applid to the peri-wound area and also aids in retention and removal. By inhibiting the lateral movement of the exudate, it prevents the maceration of the surrounding skin. Mepitel is non-absorptive but contains 1mm diameter pores that allow wound fluids to permeate into a secondary absorbent dressing [21].

SilvaSorb® [10] (Medline Industries, Mundelein, IL, USA) is a biocompatible transparent, amorphous hydrogel with a distinctive microlattice arrangement in which the scaffolding stabilises the silver ions. The dressing exerts its action for three days; therefore, daily dressing changes are not needed. The hydrogel is non-irritating or sensitising and has exceptional fluid management without being toxic to host cells or causing wound staining or pseudoeschar development [22].

Modern **silver–foam** dressings (Acticoat, Aquacel Ag, Mepilex Ag) combine the antibacterial traits of SSD with an improved mechanical barrier and absorption functions. These treatments vary significantly in their total silver concentration, ion release patterns, and therapeutic mechanism of action [22,23].

Acticoat™ [11,17–20,24,25] (Smith and Nephew, St. Petersburg, FL, USA) is a dual-layered dressing with a nanocrystalline silver coat and a polyethene layer that is bonded to a polyester core, slowly releasing small amounts of silver in an ionised form due to the humid environment. Nanocrystalline ionic silver interrupts bacterial and fungal DNA and protein synthesis. It may also disrupt the biofilm and cell wall structures although in vitro studies suggest that it might be cytotoxic to keratinocytes. Adherence to wound beds was also noted, causing potential skin-stripping and pain upon removal [26].

Aquacel® Ag [2,25] (ConvaTec Group PLC, Reading, England) is a comfortable triple-layered Hydrofiber® dressing . It contains a layer of sodium carboxymethylcellulose that forms a gel on contact with wound fluid, and it also contains antimicrobial 1.2% w/w ionic silver. The middle foam layer absorbs the wound exudate that contains the infective agents. Then, the silver exerts its bactericidal action in the fabric of the dressing without releasing the ions. Therefore, it eliminates the potential for argyria. The top sheet is water-repellent and has controlled vapour permeability and additional protection against microorganisms [27,28]. A study found that optimally, dressings should only be changed every ten days, but this approach may rarely conceal an infection due to its non-transparent design [29].

Mepilex® Ag [17–19,30,31] (Mölnlycke Health Care, Gothenburg, Sweden) consists of two layers: a soft silicone wound contact layer (Safetac®) and an absorbent polyurethane foam layer. The foam sheet contains silver and activated carbon. Dressings made from silicone adhere to intact skin and remain on the wound surface without sticking to it in situ, consequently sustaining a moist milieu and resulting in less traumatic separation [32].

Radiation-sterilised **amnion membrane (AM)** [5,33,34] allografts are biological skin substitutes and burn dressings. The human placenta's inner amnion layer is shiny and thin and is made up of cuboidal, compact epithelial cells and mesenchymal connective tissue. The thick outer chorion of the fetal membrane is composed of transitional epithelium. The placenta can be used in toto (amnion and chorion) on deep burns, or the amnion can be used alone on superficial wounds. It has low immunogenicity and contains many bioactive factors, such as collagen, cell adhesion compounds (laminins, fibronectin, proteo-, and glycosaminoglycans), and growth factors and cytokines with anti-inflammatory -bacterial, -immunogenic, and -fibrotic features [35,36].

Xenografts are temporary skin substitutes that are derived from animal or plant cells and tissues. A widely used example in tropical and subtropical regions is the **Nile tilapia** [1] (Oreochromis niloticus), which originates from the East African Nile River basin. Tilapia has a non-infectious microbiome and morphological composition that is comparable to the human skin but that has more beneficial type I collagen [37].

Biosynthetic dressings (Biobrane, EzDerm, Transcyte) contain cells or particles that originate from living organisms that have been combined or modified with modern synthetic compounds[38].

Biobrane™ [3,6,7,11,14,15,24] (Dow Hickman/ Bertek Pharmaceuticals, Sugarland, TX, USA) is a biosynthetic, bi-laminate dressing. Its made of a flexible, woven, non-biodegradable tri-filament nylon mesh with peptides derived from porcine type I collagen that have been embedded into an ultra-thin, semipermeable silicone outer film. The dressing is able to adhere firmly to the wound and spontaneously detaches when re-epithelialisation has occurred but has deficits in terms of conformability and handling; thus, it is not ideal for burns on the face and neck region. Furthermore, it does not have any specific antimicrobial qualities [39].

EZderm® [31,32,40] (Mölnlycke Health Care, Gothenburg, Sweden) is a perforated, biosynthetic porcine xenograft that is pre-meshed at a ratio of 1:1 . The collagen in this dressing

has been crosslinked with aldehydes to provide strength and durability. They can be used as a diagnostic tool for deep or infected burns, as they tend not to adhere to them, unlike superficial ones. Nevertheless, some studies have reported increased hypertrophic scarring, which is probably due to an immune response against the biological components of the dressing. Furthermore, there are cultural and ethical aspects of using pig donors and a possibility for viral transmission [41].

TransCyte® [6] (Advanced Tissue Sciences, La Jolla, CA, USA) is a translucent, temporary biosynthetic skin substitute that consists of a polymer silicone membrane bonded to a nylon mesh coated with porcine dermal collagen and neonatal human fibroblast cells cultured on that mesh in vitro. These fibroblasts secrete human dermal collagen matrix proteins and growth factors. Following freezing, the tissue matrix and growth factors are left intact when there is no metabolic activity, and after application, these particles facilitate healing until wound closure occurs [42].

ReCell® [15] (Avita Medical Ltd, Cambridge, UK) is a device that processes autologous skin samples that have been collected in an operating theatre into a suspension using a trypsin solution. Later, this solution can be applied to the wound using its Spray-On Skin system, showing comparable results to split-thickness skin grafting. ReCell can be combined with traditional dressings that may further improve patient outcomes, but the available information is controversial [43].

Adjunctive therapies (**collagenase, vitamin E+C+Zinc, wIRA, NPWT, heparin, rhGM-CSF, bFGF,** and **rhEGF**) are physical or chemical enhancers of tissue regeneration that can be added to traditional occlusive dressings to improve patient outcomes.

Collagenase [4] (Santyl® - Healthpoint Biotherapeutics, Fort Worth, TX, USA) ointment is an enzymatic debridement agent (similar to bromelain or papain [44]) that cleaves denatured collagen at seven specific sites and that creates bioactive peptides as byproducts. These particles can stimulate keratinocytes and fibroblasts, induce endothelial cell migration, and are related to the proliferative phase of reepithelialisation. It can be combined with antimicrobial agents (e.g., nystatin and polymyxin b (PMB)) to form a therapy on its own, but adding this protein to other occlusive dressings may improve outcomes, but this has yet to be investigated in children [45].

Recombinant human epidermal growth factor (rh**EGF**) [46] is a protein that is responsible for stimulating cellular proliferation, differentiation, and survival by binding to its receptor (EGFR)[47].

Basic fibroblast growth factors (**bFGF)** [48] **(**Trafermin, Fiblast Spray®, Kaken Pharmaceutical, Tokyo, Japan**)** are cell signalling proteins that have been pre-synthesised by macrophages and released at the injury site. They are often referred to as pluripotent growth factors due to their multiple types of action on many cell types, such as improving angiogenesis and keratinocyte organization [49].

Recombinant human granulocyte-macrophage colony-stimulating factor (rh**GM-CSF**) [50] is a monomeric glycoprotein that is secreted by many cells in the immune system

and that functions as a pro-inflammatory cytokine against infections. However, it also increases oedema and reactive oxygen production [51].

Heparin [52] is a naturally occurring glycosaminoglycan and anticoagulant medication that is released from mast cells directly into the vasculature. Its exact mechanism of action is unknown, but it has been proposed that its primary function is defence against foreign materials. Several side effects have been noted, including bleeding, heparin-induced thrombocytopenia, hyperkalaemia, and elevated liver enzymes. Fortunately, its impact can be counterbalanced with protamine–sulphate [53].

Vitamins E and C Supplemented with Zinc [54] have been administered due to their antioxidant traits, which may reduce the consequences of the pro-inflammatory cytokines after trauma. Moreover, vitamin C plays a vital role in collagen synthesis, and zinc is an essential part of more than 1000 transcription factors and 300 enzymes from every class [55–57].

Negative pressure wound therapy (NPWT) [12,20,58] is a non-invasive treatment that involves the controlled application of subatmospheric pressure to the wound using a dressing sealed with a transparent film connected to a vacuum pump to remove excess exudate and to promote circulation. Therefore, it creates a moist environment with reduced oedema,but it can only be used where there are no exposed blood vessels or nerves because direct foam contact may damage them [59].

Water-filtered infrared-A (wIRA) [60] produces a therapeutic heat field in the tissue created from full-spectrum light purified by a hermetically sealed water filter that blocks out all of the harmful ultraviolet light waves. Therefore, wIRA increases tissue temperature, oxygen partial pressure, and perfusion, improving energy and oxygen supply. Furthermore, it can reduce inflammation and hypersecretion as well as the need for analgesics [61].

References

1. Lima Junior, E.M.; de Moraes Filho, M.O.; Forte, A.J.; Costa, B.A.; Fechine, F.V.; Negreiros Nunes Alves, A.P.; Amaral de Moraes, M.E.; Sales Rocha, M.B.; Silva Junior, F.R.; Araujo do Nascimento Soares, M.F.; et al. Pediatric Burn Treatment Using Tilapia Skin as a Xenograft for Superficial Partial-Thickness Wounds: A Pilot Study. *J. Burn Care Res.* **2020**, *41*, 241-247, doi:10.1093/jbcr/irz149.
2. Caruso, D.M.; Foster, K.N.; Blome-Eberwein, S.A.; Twomey, J.A.; Herndon, D.N.; Luterman, A.; Silverstein, P.; Antimarino, J.R.; Bauer, G.J. Randomized clinical study of hydrofiber dressing with silver or silver sulfadiazine in the management of partial-thickness burns. *J. Burn Care Res.* **2006**, *27*, 298-309, doi:10.1097/01.BCR.0000216741.21433.66.
3. Barret, J.P.; Dziewulski, P.; Ramzy, P.I.; Wolf, S.E.; Desai, M.H.; Herndon, D.N. Biobrane versus 1% silver sulfadiazine in second-degree pediatric burns. *Plast. Reconstr. Surg.* **2000**, *105*, 62-65, doi:10.1097/00006534-200001000-00010.
4. Ostlie, D.J.; Juang, D.; Aguayo, P.; Pettiford-Cunningham, J.P.; Erkmann, E.A.; Rash, D.E.; Sharp, S.W.; Sharp, R.J.; St Peter, S.D. Topical silver sulfadiazine vs collagenase ointment for the treatment of partial thickness burns in children: a prospective randomized trial. *J. Pediatr. Surg.* **2012**, *47*, 1204-1207, doi:10.1016/j.jpedsurg.2012.03.028.
5. Mostaque, A.K.; Rahman, K.B. Comparisons of the effects of biological membrane (amnion) and silver sulfadiazine in the management of burn wounds in children. *J. Burn Care Res.* **2011**, *32*, 200-209, doi:10.1097/BCR.0b013e31820aad94.

6. Kumar, R.J.; Kimble, R.M.; Boots, R.; Pegg, S.P. Treatment of partial-thickness burns: A prospective, randomized trial using transcyte™. *ANZ J. Surg.* **2004**, *74*, 622-626, doi:10.1111/j.1445-1433.2004.03106.x.

7. Lal, S.; Barrow, R.E.; Wolf, S.E.; Chinkes, D.L.; Hart, D.W.; Heggers, J.P.; Herndon, D.N. Biobrane improves wound healing in burned children without increased risk of infection. *Shock* **2000**, *14*, 314-318; doi:10.1097/00024382-200014030-00013.

8. Gotschall, C.S.; Morrison, M.I.; Eichelberger, M.R. Prospective, randomized study of the efficacy of Mepitel on children with partial-thickness scalds. *J. Burn Care Res.* **1998**, *19*, 279-283, doi:10.1097/00004630-199807000-00002.

9. Bugmann, P.; Taylor, S.; Gyger, D.; Lironi, A.; Genin, B.; Vunda, A.; La Scala, G.; Birraux, J.; Le Coultre, C. A silicone-coated nylon dressing reduces healing time in burned paediatric patients in comparison with standard sulfadiazine treatment: a prospective randomized trial. *Burns* **1998**, *24*, 609-612, doi:10.1016/s0305-4179(98)00095-3.

10. Glat, P.M.; Kubat, W.D.; Hsu, J.F.; Copty, T.; Burkey, B.A.; Davis, W.; Goodwin, I. Randomized Clinical Study of SilvaSorb (R) Gel in Comparison to Silvadene (R) Silver Sulfadiazine Cream in the Management of Partial-Thickness Burns. *J. Burn Care Res.* **2009**, *30*, 262-267, doi:10.1097/BCR.0b013e318198a2e8.

11. Muganza, A.; Cullingworth, L. A Prospective Single-Centre Randomised Controlled Study to Compare the Time to Healing of Partial Thickness Burn Wounds Treated with Versajet, Biobrane and Acticoat to Conventional Therapy. *Glob. J. Med. Res.* **2015**. 14. Available online: https://medicalresearchjournal.org/index.php/GJMR/article/view/865/775 (accessed on 8 November 2020).

12. Zheng, X.P.; Chen, J.; Chen, T.S.; Jiang, Y.N.; Shen, T.; Xiao, S.C.; Hu, X.Y. Preliminary effect observation on the application of micro-negative pressure in children with small-area deep partial-thickness burn. *Zhonghua shaoshang zazhi = Chin. J. Burn.* **2019**, *35*, 720-725, doi:10.3760/cma.j.issn.1009-2587.2019.10.004.

13. Oaks, R.J.; Cindass, R. Silver Sulfadiazine. In *StatPearls*; StatPearls Publishing LLC.: Treasure Island (FL), 2021. PMID: 32310514.

14. Cassidy, C.; Peter, S.D.S.; Lacey, S.; Beery, M.; Ward-Smith, P.; Sharp, R.J.; Ostlie, D.J. Biobrane versus Duoderm for the treatment of intermediate thickness burns in children: A prospective, randomized trial. *Burns* **2005**, *31*, 890-893, doi:10.1016/j.burns.2005.04.029.

15. Wood, F.; Martin, L.; Lewis, D.; Rawlins, J.; McWilliams, T.; Burrows, S.; Rea, S. A prospective randomised clinical pilot study to compare the effectiveness of Biobrane® synthetic wound dressing, with or without autologous cell suspension, to the local standard treatment regimen in paediatric scald injuries. *Burns* **2012**, *38*, 830-839, doi:10.1016/j.burns.2011.12.020.

16. Singh, A.; Halder, S.; Chumber, S.; Misra, M.C.; Sharma, L.K.; Srivastava, A.; Menon, G.R. Meta-analysis of Randomized Controlled Trials on Hydrocolloid Occlusive Dressing Versus Conventional Gauze Dressing in the Healing of Chronic Wounds. *Asian J. Surg.* **2004**, *27*, 326-332, doi:10.1016/S1015-9584(09)60061-0.

17. Gee Kee, E.L.; Kimble, R.M.; Cuttle, L.; Khan, A.; Stockton, K.A. Randomized controlled trial of three burns dressings for partial thickness burns in children. *Burns* **2015**, *41*, 946-955, doi:10.1016/j.burns.2014.11.005.

18. Gee Kee, E.L.; Stockton, K.A.; Kimble, R.M.; Cuttle, L.; McPhail, S.M. Cost-effectiveness of silver dressings for paediatric partial thickness burns: An economic evaluation from a randomized controlled trial. *Burns* **2017**, *43*, 724-732, doi:10.1016/j.burns.2016.09.018.

19. Gee Kee, E.L.; Kimble, R.M.; Cuttle, L.; Stockton, K.A. Scar outcome of children with partial thickness burns: A 3 and 6 month follow up. *Burns* **2016**, *42*, 97-103, doi:10.1016/j.burns.2015.06.019.

20. Frear, C.C.; Cuttle, L.; McPhail, S.M.; Chatfield, M.D.; Kimble, R.M.; Griffin, B.R. Randomized clinical trial of negative pressure wound therapy as an adjunctive treatment for small-area thermal burns in children. *Br. J. Surg.* **2020**, doi:10.1002/bjs.11993.

21. Williams, C. Mepitel. *Br. J. Nurs.* **1995**, *4*, 51-55, doi:10.12968/bjon.1995.4.1.51.

22. Castellano, J.J.; Shafii, S.M.; Ko, F.; Donate, G.; Wright, T.E.; Mannari, R.J.; Payne, W.G.; Smith, D.J.; Robson, M.C. Comparative evaluation of silver-containing antimicrobial dressings and drugs. *Int. Wound J.* **2007**, *4*, 114-122, doi:10.1111/j.1742-481X.2007.00316.x.

23. Chaganti, P.; Gordon, I.; Chao, J.H.; Zehtabchi, S. A systematic review of foam dressings for partial thickness burns. *Am. J. Emerg. Med.* **2019**, *37*, 1184-1190, doi:10.1016/j.ajem.2019.04.014.

24. Hyland, E.J.; D'Cruz, R.; Menon, S.; Harvey, J.G.; La Hei, E.; Lawrence, T.; Waddell, K.; Nash, M.; Holland, A.J.A. Biobrane™ versus acticoat™ for the treatment of mid-dermal pediatric burns: A prospective randomized controlled pilot study. *Int. J. Burn. Trauma* **2018**, *8*, 63-67.

25. Brown, M.; Dalziel, S.R.; Herd, E.; Johnson, K.; She, R.W.; Shepherd, M. A Randomized Controlled Study of Silver-Based Burns Dressing in a Pediatric Emergency Department. *J. Burn Care Res.* **2016**, *37*, E340-E347, doi:10.1097/bcr.0000000000000273.

26. Dunn, K.; Edwards-Jones, V. The role of Acticoat™ with nanocrystalline silver in the management of burns. *Burns* **2004**, *30*, S1-S9, doi:10.1016/S0305-4179(04)90000-9.

27. Richetta, A.G.; Cantisani, C.; Li, V.W.; Mattozzi, C.; Melis, L.; De Gado, F.; Giancristoforo, S.; Silvestri, E.; Calvieri, S. Hydrofiber dressing and wound repair: review of the literature and new patents. *Recent Pat. Inflamm. Allergy Drug Discov.* **2011**, *5*, 150-154, doi:10.2174/187221311795399264.

28. Barnea, Y.; Weiss, J.; Gur, E. A review of the applications of the hydrofiber dressing with silver (Aquacel Ag) in wound care. *Ther. Clin. Risk Manag.* **2010**, *6*, 21-27.

29. Demircan, M.; Yetis, M.I.; Cicek, T.; Bayrakci, E.; Tasci, A. Comparison of five different times for change of a silver-containing soft silicone foam dressing in pediatric partial thickness burns. *J. Burn Care Res.* **2015**, *36*, S193, doi:10.1097/BCR.0000000000000251.

30. Karlsson, M.; Steinvall, I.; Sjoberg, F.; Olofsson, P.; Elmasry, M. Burn scar outcome at six and 12 months after injury in children with partial thickness scalds: Effects of dressing treatment. *Burns* **2020**, *46*, 546-551, doi:10.1016/j.burns.2020.02.007.

31. Karlsson, M.; Elmasry, M.; Steinvall, I.; Sjoberg, F.; Olofsson, P.; Thorfinn, J. Superiority of silver-foam over porcine xenograft dressings for treatment of scalds in children: A prospective randomised controlled trial. *Burns* **2019**, *45*, 1401-1409, doi:10.1016/j.burns.2019.04.004.

32. Davies, P.; McCarty, S.; Hamberg, K. Silver-containing foam dressings with Safetac: a review of the scientific and clinical data. *J. Wound Care* **2017**, *26*, S1-s32, doi:10.12968/jowc.2017.26.Sup6a.S1.

33. Omranifard, M.; Ansari, M.; Jazebi, N.; Rouzbahani, R.; Akbari, M. The comparison between long-term results of treatment with amnion graft and skin graft in children second degree burn wounds. *J. Isfahan Med. Sch.* **2011**, *29*.

34. Branski, L.K.; Herndon, D.N.; Celis, M.M.; Norbury, W.B.; Masters, O.E.; Jeschke, M.G. Amnion in the treatment of pediatric partial-thickness facial burns. *Burns* **2008**, *34*, 393-399, doi:10.1016/j.burns.2007.06.007.

35. Joshi, C.J.; Hassan, A.; Carabano, M.; Galiano, R.D. Up-to-date role of the dehydrated human amnion/chorion membrane (AMNIOFIX) for wound healing. *Expert Opin. Biol. Ther.* **2020**, *20*, 1125-1131, doi:10.1080/14712598.2020.1787979.

36. Palanker, N.D.; Lee, C.T.; Weltman, R.L.; Tribble, G.D.; van der Hoeven, R.; Hong, J.; Wang, B. Antimicrobial Efficacy Assessment of Human Derived Composite Amnion-Chorion Membrane. *Sci. Rep.* **2019**, *9*, 15600, doi:10.1038/s41598-019-52150-4.

37. Osama, M. Use of Nile Tilapia (Oreochromisniloticus) skin in the management of skin burns. *J. Pak. Med. Assoc.* **2017**, *67*, 1955.

38. Wasiak, J.; Cleland, H. Burns: dressings. *BMJ Clin. Evid.* **2015**, *2015*.

39. Whitaker, I.S.; Prowse, S.; Potokar, T.S. A Critical Evaluation of the Use of Biobrane as a Biologic Skin Substitute: A Versatile Tool for the Plastic and Reconstructive Surgeon. *Ann. Plast. Surg.* **2008**, *60*, 333-337, doi:10.1097/SAP.0b013e31806bf446.

40. Healy, C.M.; Boorman, J.G. Comparison of E-Z Derm and Jelonet dressings for partial skin thickness burns. *Burns* **1989**, *15*, 52-54, doi:10.1016/0305-4179(89)90072-7.

41. Troy, J.; Karlnoski, R.; Downes, K.; Brown, K.S.; Cruse, C.W.; Smith, D.J.; Payne, W.G. The Use of EZ Derm® in Partial-Thickness Burns: An Institutional Review of 157 Patients. *Eplasty* **2013**, *13*, e14-e14.

42. Lukish, J.R.; Eichelberger, M.R.; Newman, K.D.; Pao, M.; Nobuhara, K.; Keating, M.; Golonka, N.; Pratsch, G.; Misra, V.; Valladares, E.; et al. The use of a bioactive skin substitute decreases length of stay for pediatric burn patients. *J. Pediatr. Surg.* **2001**, *36*, 1118-1121, doi:10.1053/jpsu.2001.25678.

43. Peirce, S.C.; Carolan-Rees, G. ReCell(®) Spray-On Skin System for Treating Skin Loss, Scarring and Depigmentation after Burn Injury: A NICE Medical Technology Guidance. *Appl. Health Econ. Health Policy* **2019**, *17*, 131-141, doi:10.1007/s40258-018-00457-0.

44. Hirche, C.; Kreken Almeland, S.; Dheansa, B.; Fuchs, P.; Governa, M.; Hoeksema, H.; Korzeniowski, T.; Lumenta, D.B.; Marinescu, S.; Martinez-Mendez, J.R.; et al. Eschar removal by bromelain based enzymatic debridement (Nexobrid®) in burns: European consensus guidelines update. *Burns* **2020**, *46*, 782-796, doi:10.1016/j.burns.2020.03.002.

45. Riley, K.N.; Herman, I.M. Collagenase promotes the cellular responses to injury and wound healing in vivo. *J. Burns Wounds* **2005**, *4*, e8-e8.

46. Liang, Z.Q.; Li, H.M.; Meng, C.Y. Repair of second degree facial burns in children using recombinant human epidermal growth factor. *J. Clin. Rehabil. Tissue Eng. Res.* **2007**, *11*, 1974-1975.

47. Kaya, O.; Orhan, E.; Sapmaz-Metin, M.; Topçu-Tarladaçalışır, Y.; Gündüz, Ö.; Aydın, B. The effects of epidermal growth factor on early burn-wound progression in rats. *Dermatol. Ther.* **2020**, *33*, e13196, doi:10.1111/dth.13196.

48. Hayashida, K.; Akita, S. Quality of pediatric second-degree burn wound scars following the application of basic fibroblast growth factor: Results of a randomized' controlled pilot study. *Ostomy Wound Manag.* **2012**, *58*, 32-36.

49. Gibran, N.S.; Isik, F.F.; Heimbach, D.M.; Gordon, D. Basic fibroblast growth factor in the early human burn wound. *J. Surg. Res.* **1994**, *56*, 226-234, doi:10.1006/jsre.1994.1036.

50. Jiaao, Y.; Kai, S.; Hua, J.Z.; Chun, Z.J.; Hai, N.Z.; Jing, L.H.; Wei, W.W.; Qiang, L.J. Treatment of deep second-degree facial burns in pediatric population with recombinant human GM-CSF hydrogel. *Wound Repair Regen.* **2011**, *19*, A29, doi:10.1111/j.1524-475X.2010.00670.x.

51. Hu, X.; Sun, H.; Han, C.; Wang, X.; Yu, W. Topically applied rhGM-CSF for the wound healing: a systematic review. *Burns* **2011**, *37*, 729-741, doi:10.1016/j.burns.2010.08.016.

52. Venkatachalapathy, T.S. A Comparative Study of Paediatric Thermal Burns Treated with Topical Heparin and Without Heparin. *Indian J. Surg.* **2014**, *76*, 282-287, doi:10.1007/s12262-012-0674-6.

53. Oremus, M.; Hanson, M.; Whitlock, R.; Young, E.; Gupta, A.; Dal Cin, A.; Archer, C.; Raina, P. The uses of heparin to treat burn injury. *Evid. Rep. Technol. Assess.* **2006**, 1-58.

54. Barbosa, E.; Faintuch, J.; Machado Moreira, E.A.; Goncalves da Silva, V.R.; Lopes Pereima, M.J.; Martins Fagundes, R.L.; Wilhelm Filho, D. Supplementation of Vitamin E, Vitamin C, and Zinc Attenuates Oxidative Stress in Burned Children: A Randomized, Double-Blind, Placebo-Controlled Pilot Study. *J. Burn Care Res.* **2009**, *30*, 859-866, doi:10.1097/BCR.0b013e3181b487a8.

55. Rizzo, J.A.; Rowan, M.P.; Driscoll, I.R.; Chung, K.K.; Friedman, B.C. Vitamin C in Burn Resuscitation. *Crit. Care Clin.* **2016**, *32*, 539-546, doi:10.1016/j.ccc.2016.06.003.

56. Butt, H.; Mehmood, A.; Ali, M.; Tasneem, S.; Tarar, M.N.; Riazuddin, S. Vitamin E preconditioning alleviates in vitro thermal stress in cultured human epidermal keratinocytes. *Life Sci.* **2019**, *239*, 116972, doi:10.1016/j.lfs.2019.116972.

57. Lin, P.H.; Sermersheim, M.; Li, H.; Lee, P.H.U.; Steinberg, S.M.; Ma, J. Zinc in Wound Healing Modulation. *Nutrients* **2017**, *10*, doi:10.3390/nu10010016.

58. Shen, C.A.; Chai, J.K.; Tuo, X.Y.; Cai, J.H.; Li, D.J.; Zhang, L.; Zhu, H.; Cai, J.D. Efficacy observation on application of negative pressure therapy in the treatment of superficial partial-thickness scald wound in

children. *Zhonghua Shaoshang Zazhi = Chin. J. Burn.* **2013**, *29*, 14-17, doi:10.3760/cma.j.issn.1009-2587.2013.01.005.

59. Mendonca, D.A.; Papini, R.; Price, P.E. Negative-pressure wound therapy: a snapshot of the evidence. *Int. Wound J.* **2006**, *3*, 261-271, doi:10.1111/j.1742-481X.2006.00266.x.

60. Hartel, M.; Illing, P.; Mercer, J.B.; Lademann, J.; Daeschlein, G.; Hoffmann, G. Therapy of acute wounds with water-filtered infrared-A (wIRA). *GMS Krankenhaushy. Interdiszip.* **2007**, *2*, Doc53-Doc53.

61. Hoffmann, G.; Hartel, M.; Mercer, J.B. Heat for wounds - water-filtered infrared-A (wIRA) for wound healing - a review. *Ger. Med. Sci.* **2016**, *14*, Doc08, doi:10.3205/000235.
