# Supplementary material for: Paediatric Partial-Thickness Burn Therapy: A Meta-Analysis and Systematic Review of Randomised Controlled Trials
Source: Life (Basel). 2022 Apr 21;12(5):619. doi: 10.3390/life12050619 (PMC9144506; doi:10.3390/life12050619)
Supplement: Supplementary file 1 [file life-12-00619-s001.zip › STexts/S2Text.pdf]

S2 Text: Search keys.

Search key for the Embase database

('child'/exp OR child OR 'child' OR 'children' OR 'infant'/exp OR infant OR 'infant' OR 'newborn'/exp OR newborn OR 'neonate' OR 'baby'/exp OR baby OR 'toddler'/exp OR toddler OR 'adolescent'/exp OR adolescent OR 'adolescent' OR 'teenager' OR 'pediatric'/exp OR pediatric OR 'paediatric'/exp OR paediatric) AND ('burn'/exp OR burn OR 'scald'/exp OR scald OR 'thermal injury'/exp OR 'thermal injury' OR 'flame'/exp OR flame) AND ('partial thickness' OR 'second degree' OR 'deep partial' OR 'superficial partial' OR 'mid dermal' OR 'intermediate thickness')

Search key for the CENTRAL, MEDLINE (via PubMed) and Web of Science databases

((child* OR pediatric OR paediatric OR infant* OR newborn* OR baby* OR adolescent* OR teenager*) AND (burn* OR scald* OR flame* OR thermal injur*) AND (second degree* OR partial thickness* OR deep partial* OR superficial partial* OR mid dermal* OR intermediate thickness*))
