# Supplementary figures and images for: Paediatric Partial-Thickness Burn Therapy: A Meta-Analysis and Systematic Review of Randomised Controlled Trials
Source: Life (Basel). 2022 Apr 21;12(5):619. doi: 10.3390/life12050619 (PMC9144506; doi:10.3390/life12050619)

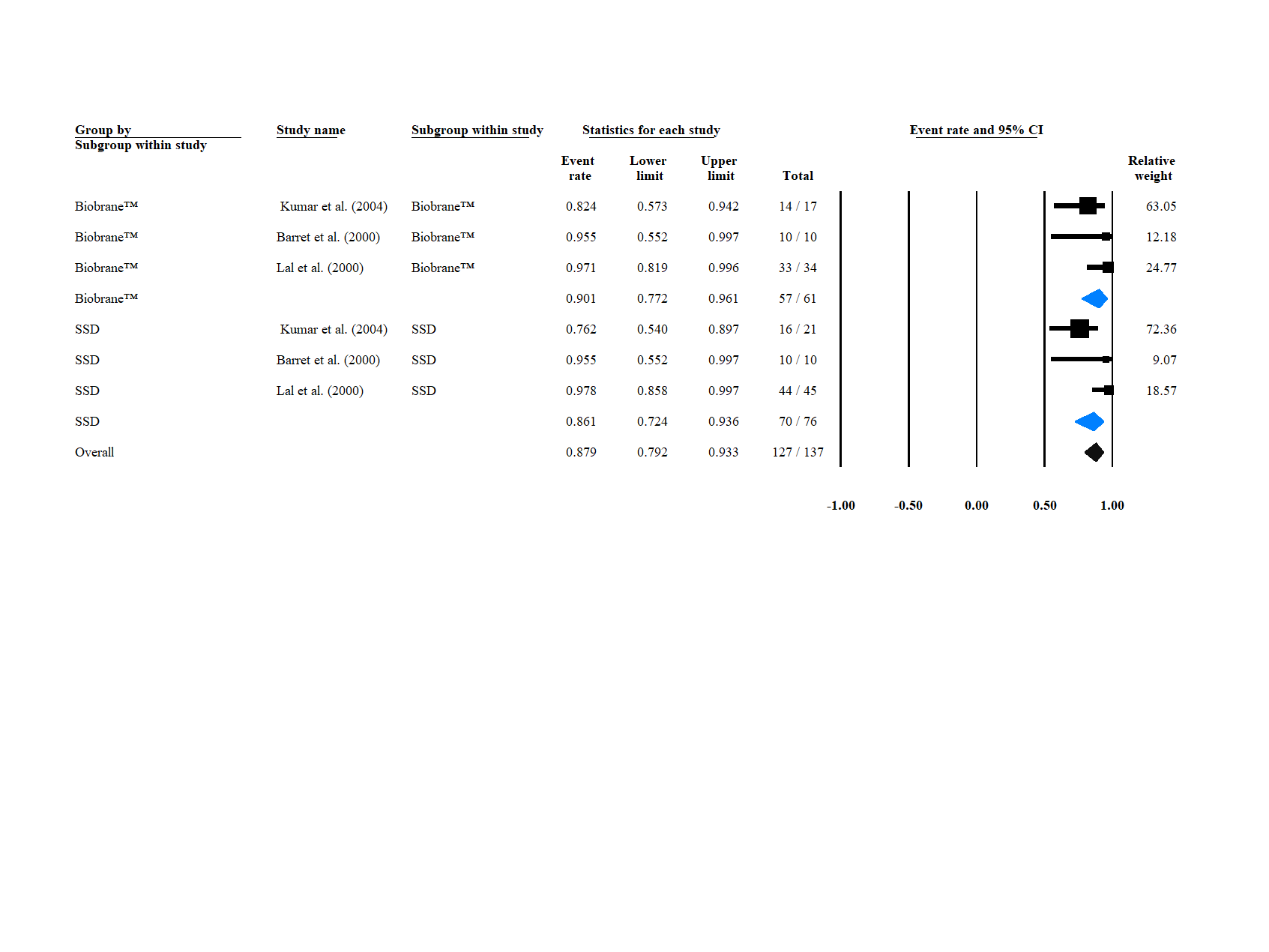

Supplement: Supplementary file 1 [file life-12-00619-s001.zip › SFigs/S10Fig.tif]

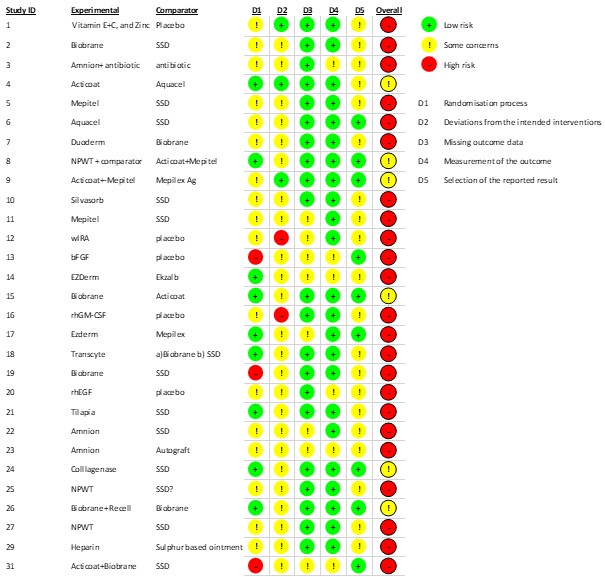

Supplement: Supplementary file 1 [file life-12-00619-s001.zip › SFigs/S1Fig.jpg]

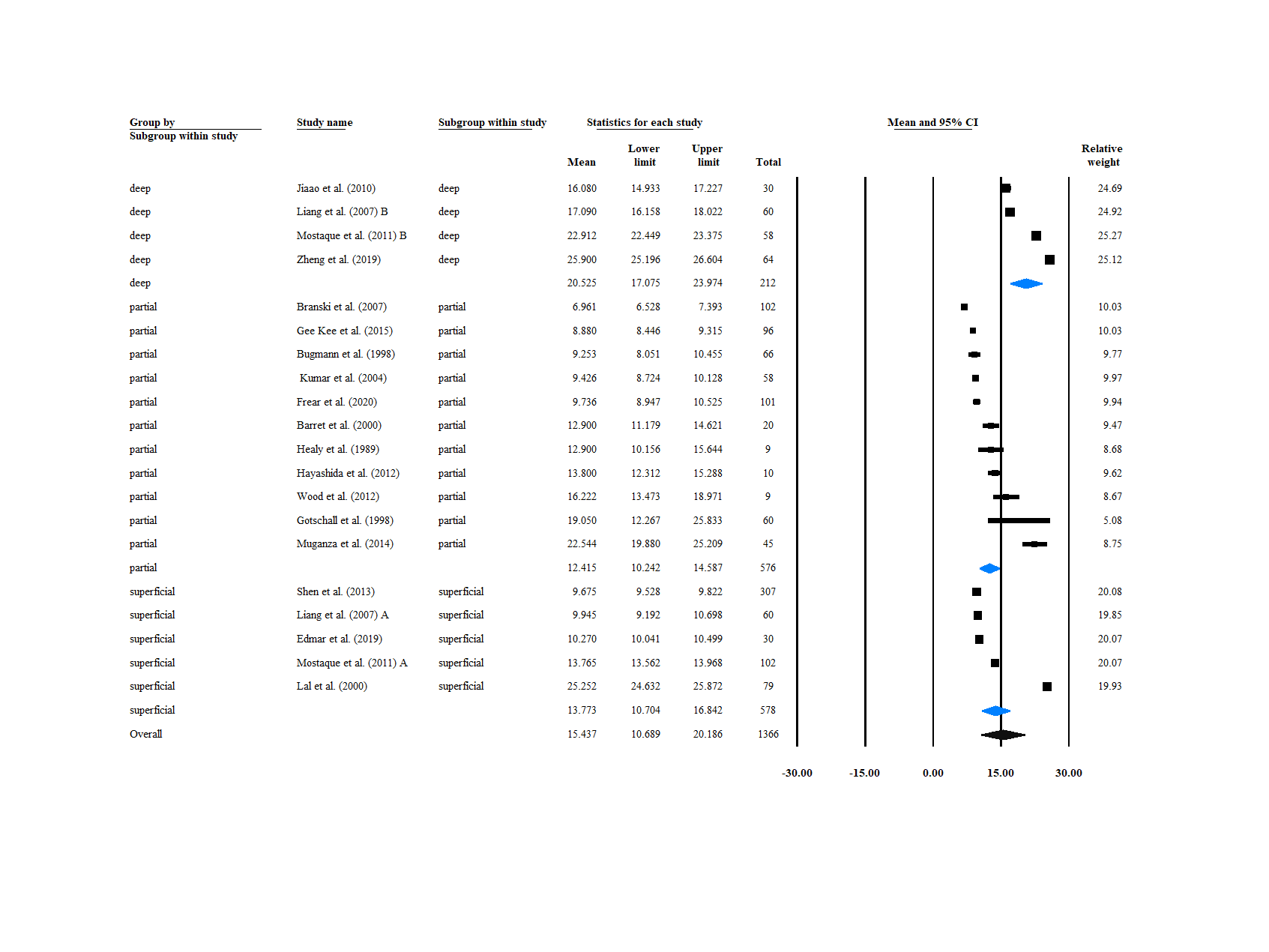

Supplement: Supplementary file 1 [file life-12-00619-s001.zip › SFigs/S2Fig.tif]

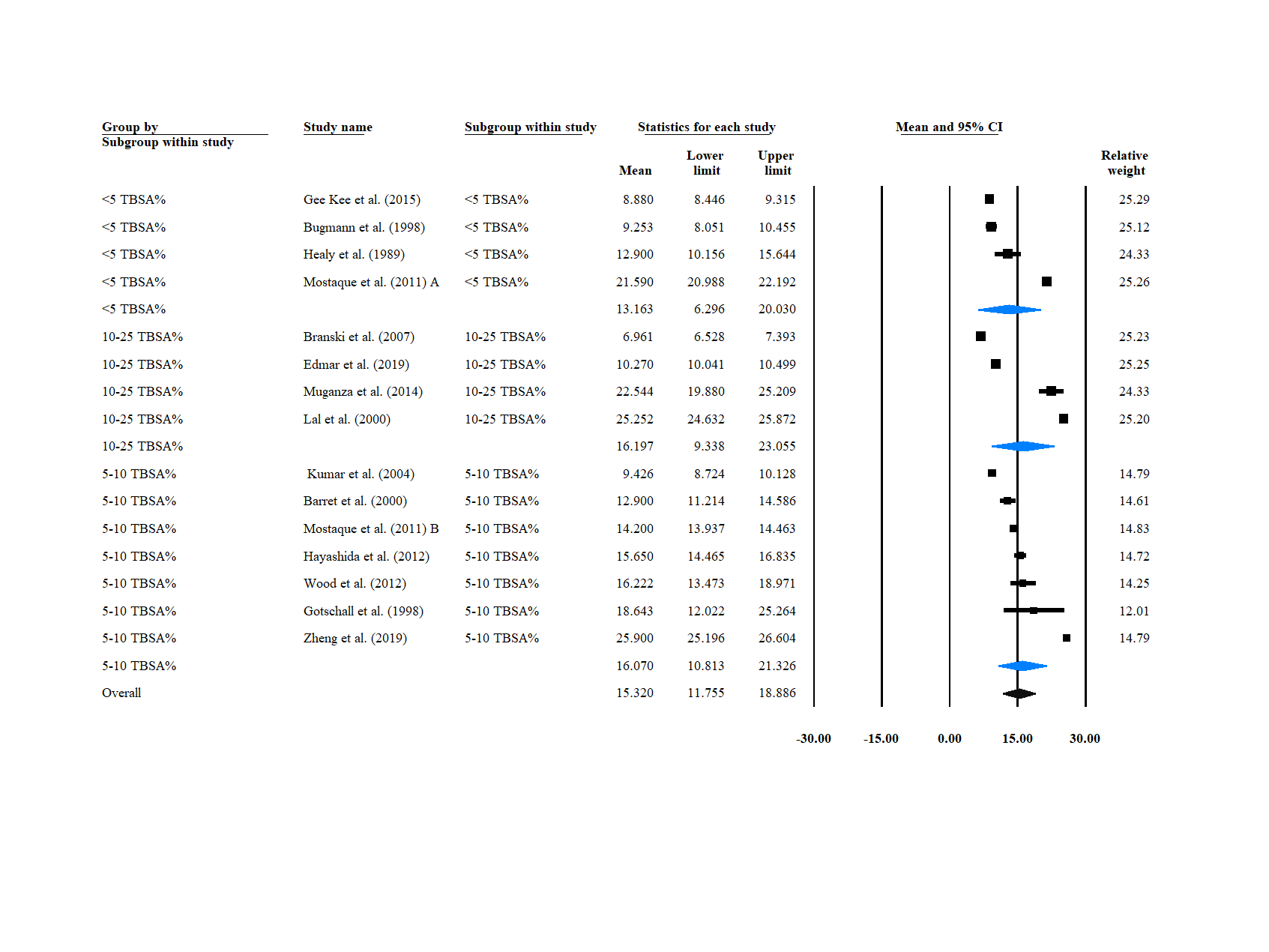

Supplement: Supplementary file 1 [file life-12-00619-s001.zip › SFigs/S3Fig.tif]

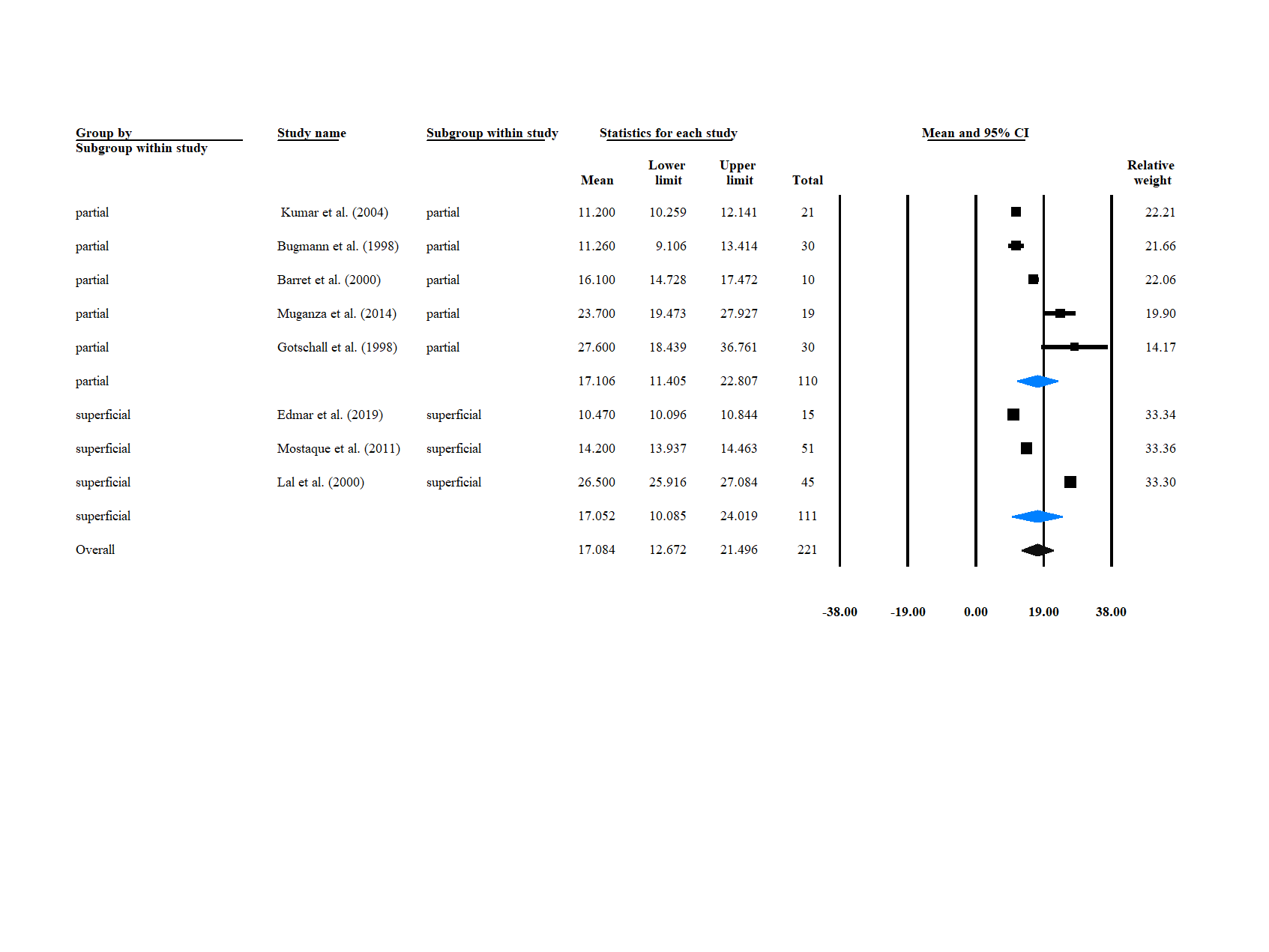

Supplement: Supplementary file 1 [file life-12-00619-s001.zip › SFigs/S4Fig.tif]

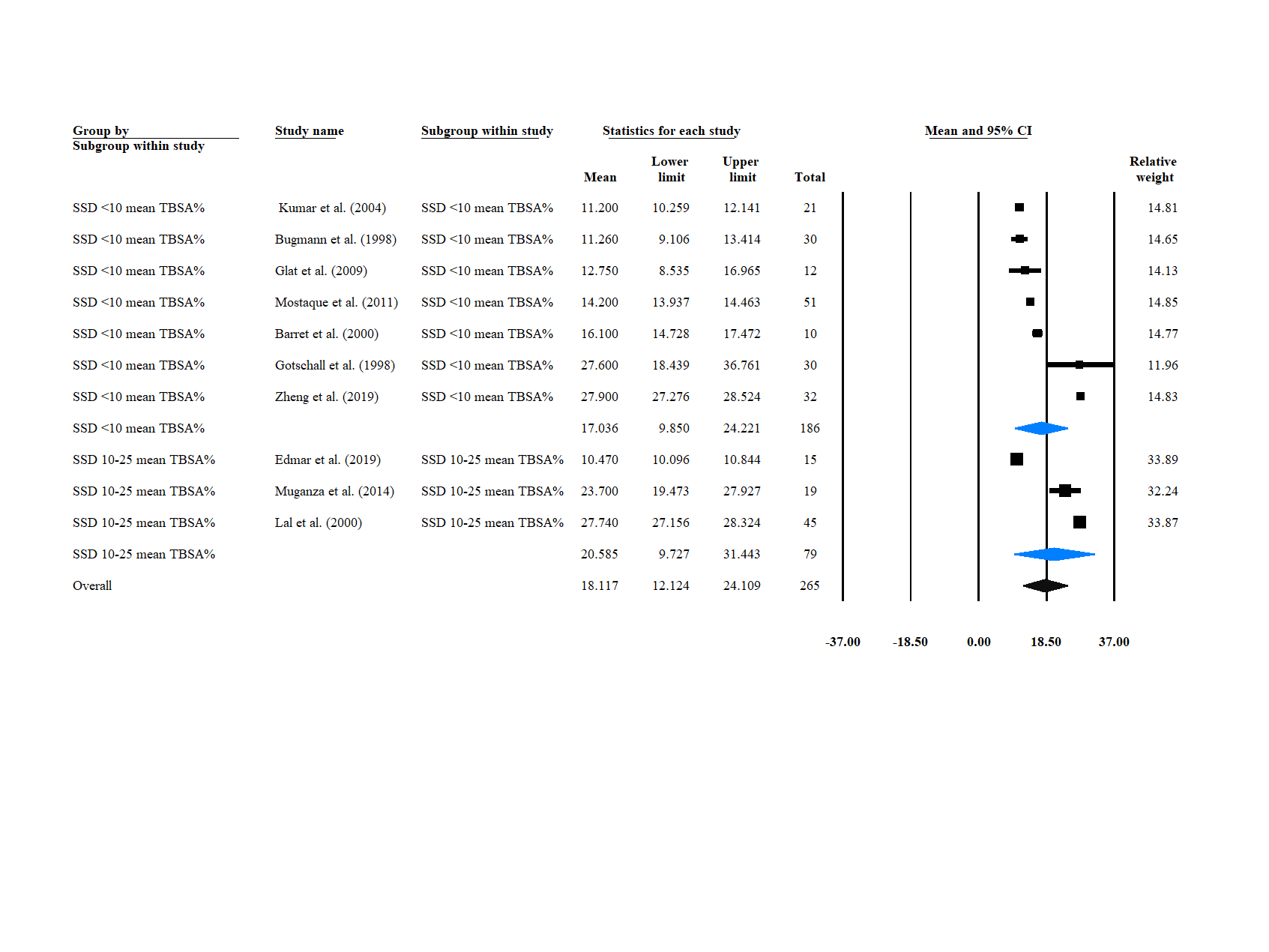

Supplement: Supplementary file 1 [file life-12-00619-s001.zip › SFigs/S5Fig.tif]

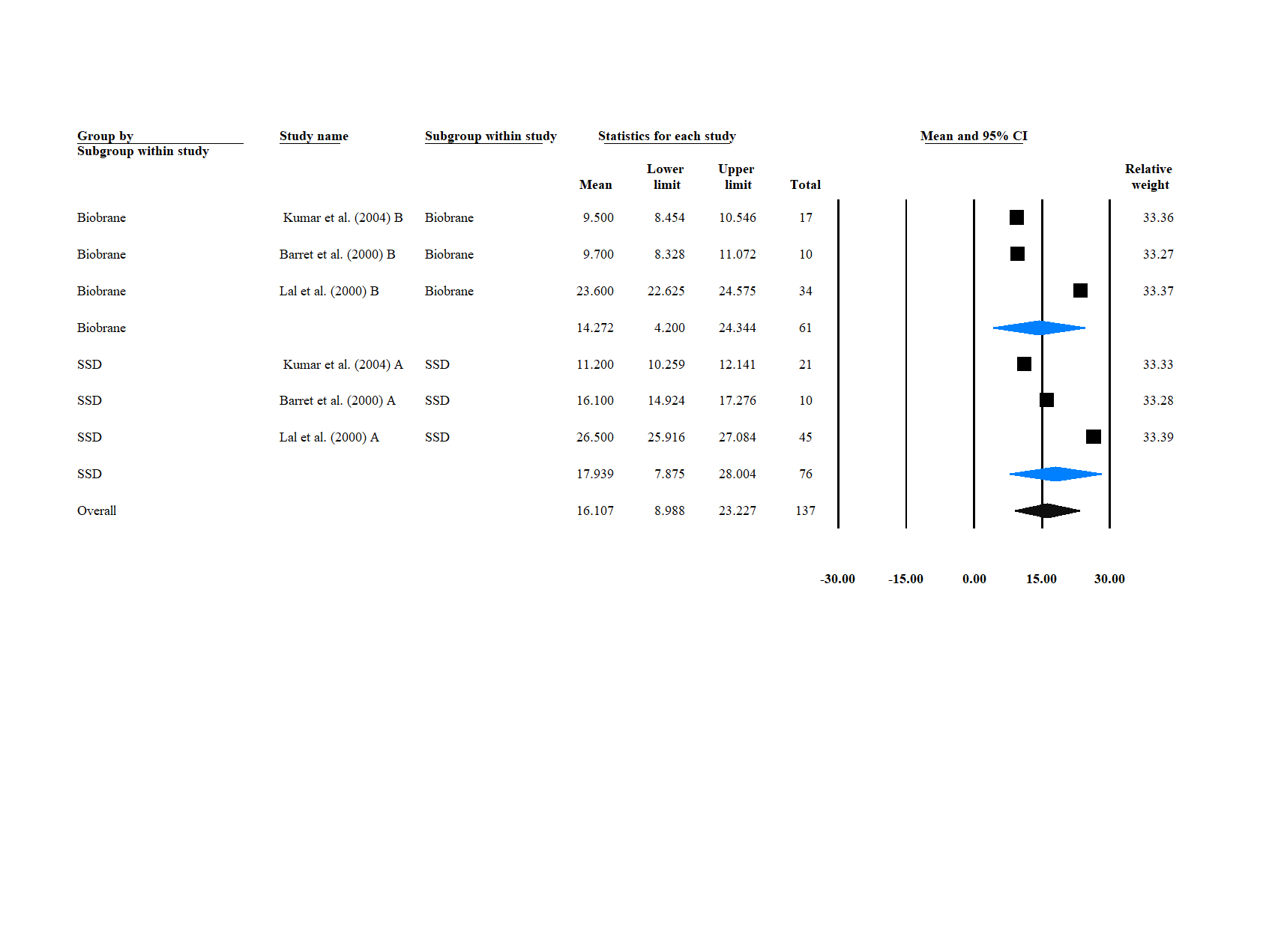

Supplement: Supplementary file 1 [file life-12-00619-s001.zip › SFigs/S6Fig.tif]

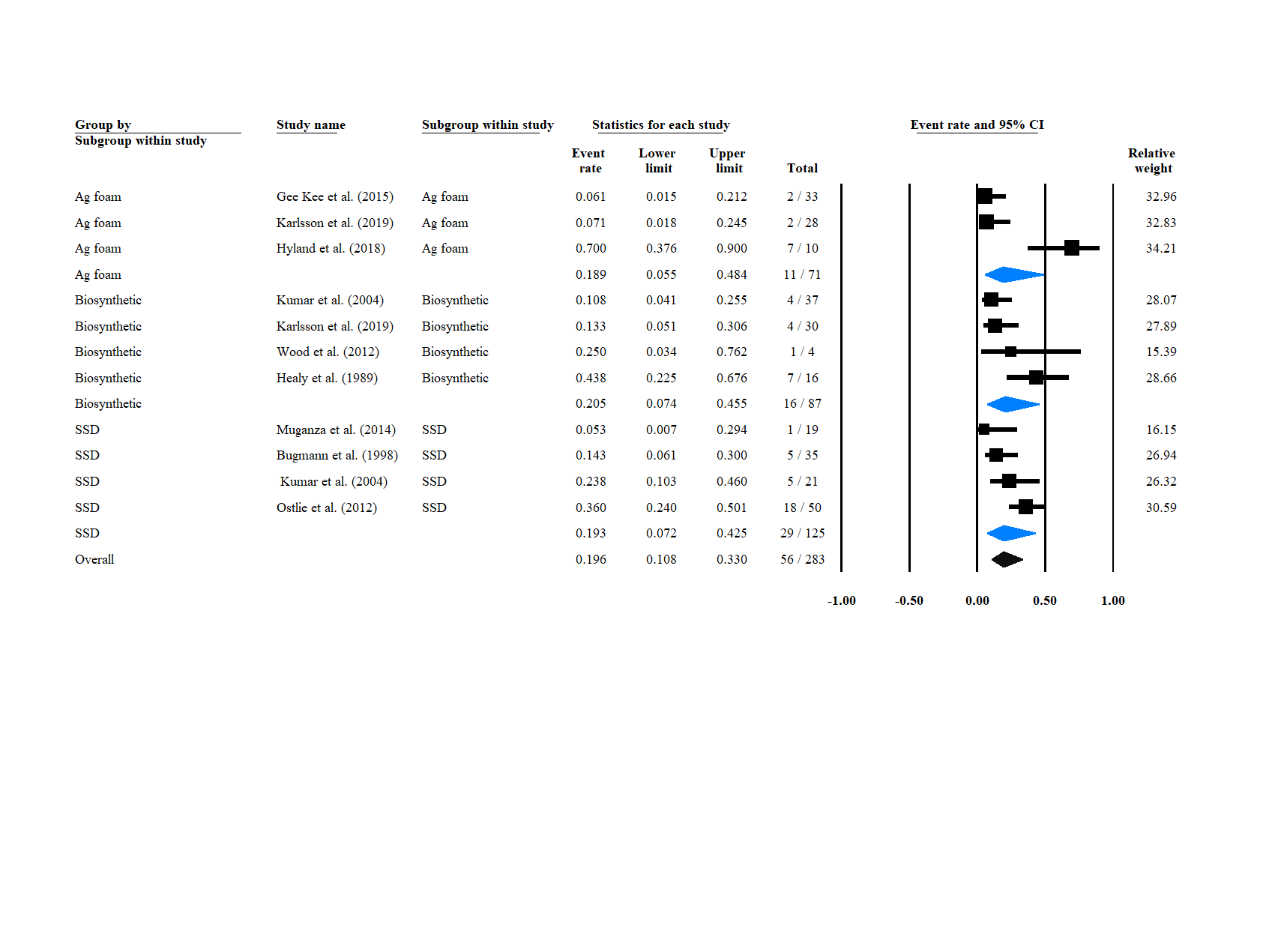

Supplement: Supplementary file 1 [file life-12-00619-s001.zip › SFigs/S7Fig.tif]

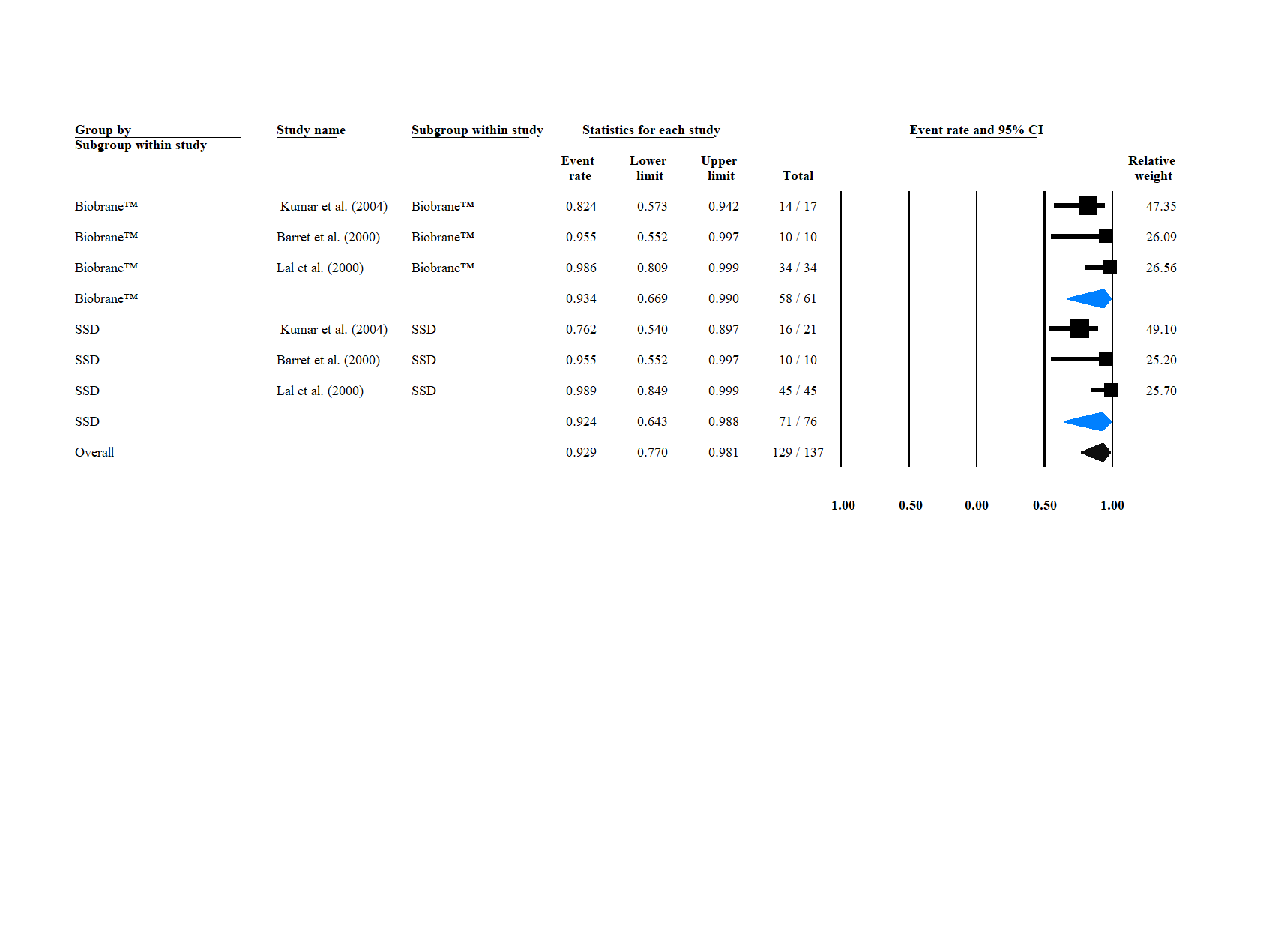

Supplement: Supplementary file 1 [file life-12-00619-s001.zip › SFigs/S8Fig.tif]

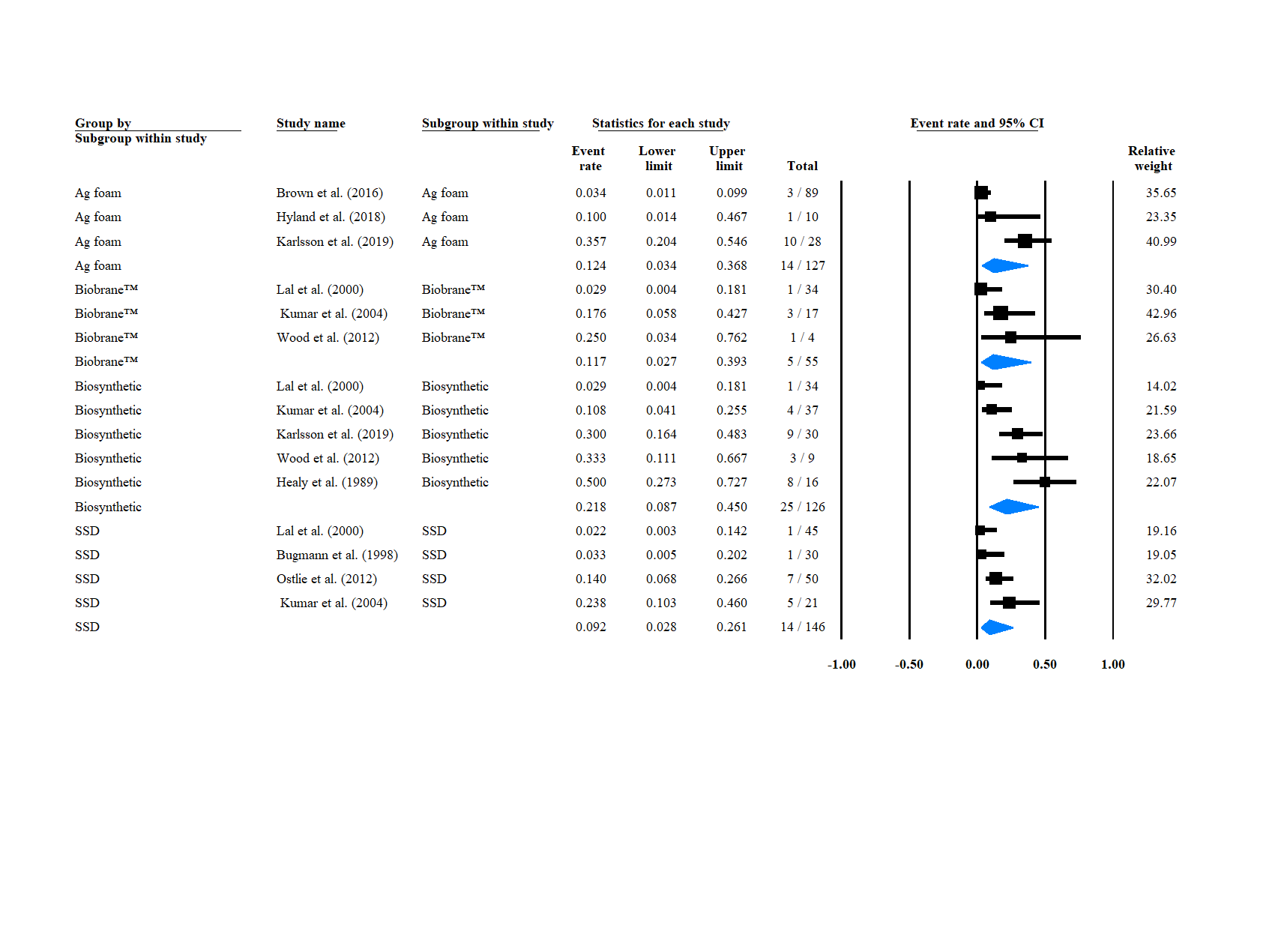

Supplement: Supplementary file 1 [file life-12-00619-s001.zip › SFigs/S9Fig.tif]
